# Two-headed UNetEfficientNets for parallel execution of segmentation and classification of brain tumors: incorporating postprocessing techniques with connected component labelling

**DOI:** 10.1007/s00432-024-05718-1

**Published:** 2024-04-29

**Authors:** Hari Mohan Rai, Joon Yoo, Serhii Dashkevych

**Affiliations:** 1 School of Computing, Gachon University, 1342 Seongnam-daero, Sujeong-Gu, Seongnam-Si, 13120 Gyeonggi-Do Republic of Korea; 2 Department of Computer Engineering, Vistula University, Stokłosy 3, 02-787 Warszawa, Poland

**Keywords:** Brain tumor, Classification, Segmentation, UNetEfficientNet, Magnetic resonance imaging, Postprocessing

## Abstract

**Purpose:**

The purpose of this study is to develop accurate and automated detection and segmentation methods for brain tumors, given their significant fatality rates, with aggressive malignant tumors like Glioblastoma Multiforme (GBM) having a five-year survival rate as low as 5 to 10%. This underscores the urgent need to improve diagnosis and treatment outcomes through innovative approaches in medical imaging and deep learning techniques.

**Methods:**

In this work, we propose a novel approach utilizing the two-headed UNetEfficientNets model for simultaneous segmentation and classification of brain tumors from Magnetic Resonance Imaging (MRI) images. The model combines the strengths of EfficientNets and a modified two-headed Unet model. We utilized a publicly available dataset consisting of 3064 brain MR images classified into three tumor classes: Meningioma, Glioma, and Pituitary. To enhance the training process, we performed 12 types of data augmentation on the training dataset. We evaluated the methodology using six deep learning models, ranging from UNetEfficientNet-B0 to UNetEfficientNet-B5, optimizing the segmentation and classification heads using binary cross entropy (BCE) loss with Dice and BCE with focal loss, respectively. Post-processing techniques such as connected component labeling (CCL) and ensemble models were applied to improve segmentation outcomes.

**Results:**

The proposed UNetEfficientNet-B4 model achieved outstanding results, with an accuracy of 99.4% after postprocessing. Additionally, it obtained high scores for DICE (94.03%), precision (98.67%), and recall (99.00%) after post-processing. The ensemble technique further improved segmentation performance, with a global DICE score of 95.70% and Jaccard index of 91.20%.

**Conclusion:**

Our study demonstrates the high efficiency and accuracy of the proposed UNetEfficientNet-B4 model in the automatic and parallel detection and segmentation of brain tumors from MRI images. This approach holds promise for improving diagnosis and treatment planning for patients with brain tumors, potentially leading to better outcomes and prognosis.

**Supplementary Information:**

The online version contains supplementary material available at 10.1007/s00432-024-05718-1.

## Introduction

**Fig. 1:**
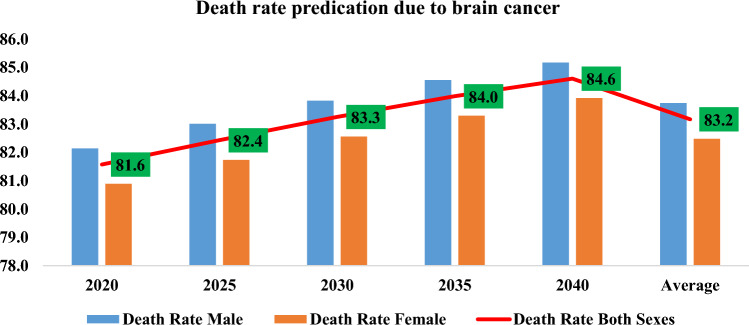
Estimation of global death rate from brain tumor between year 2020–2023

Brain tumors pose a significant global health challenge, contributing to the overall burden of cancer worldwide. In 2018, the global mortality rate due to cancer was approximately 9.6 million, making it the second leading cause of death globally, following cardiovascular diseases. Alarmingly, around 70% of these cancer-related deaths occurred in low- and middle-income countries (WHO 2020). Among the diverse array of cancer types, some of the most common ones include lung, stomach, liver, brain, prostate, breast, and thyroid cancers. While all types of tumors are concerning, brain tumors hold a particularly ominous reputation due to their life-threatening nature. Glioblastoma Multiforme (GBM) is the most common and aggressive malignant brain tumor. The five-year survival rate for GBM is generally low, ranging from 5 to 10%. The median overall survival for GBM is around 12 to 15 months. This underscores the urgent need for early detection, effective treatment, and improved management strategies for brain tumors. The prevalence of brain tumors is substantial worldwide. In the United States alone, it is estimated that around 700,000 people are currently living with brain tumors as of 2018. Furthermore, India reports a significant number of cases annually, with approximately 40,000 to 50,000 new cases diagnosed each year India (The Indian Express 2019; NBTS 2020). These values highlight the magnitude of the issue and the profound impact brain tumors have on individuals and healthcare systems. It is important to differentiate between primary and secondary brain tumors. Primary brain tumors originate in the brain itself, accounting for around 70% of cases. In contrast, secondary brain tumors, also known as metastatic tumors, occur when cancer spreads from other parts of the body to the brain. This distinction is crucial as it affects treatment approaches and prognosis. Looking ahead, projections indicate that the United States will witness approximately 87,000 new cases of primary brain tumors by the year 2020 (NBTS 2020). The projected number of deaths caused by brain tumors for male, female, and both sexes combined from the years 2020–2040 is illustrated in Fig. 1. The data highlights the significant impact of brain tumors on mortality rates, with the combined death rate exceeding 81%. This emphasizes the severity and fatality of this disease, necessitating further research and efforts to improve prevention, early detection, and treatment strategies.

Tumors are abnormal growths of cells that can occur in various parts of the body. They arise from the uncontrolled proliferation of cells, leading to the formation of a mass or lump. Tumors can develop in different tissues or organs, such as the brain, breast, lung, colon, skin, or prostate. Benign brain tumors are non-cancerous growths that usually have clear borders and do not invade surrounding tissues. They tend to grow slowly and are typically localized to a specific area of the brain. Examples of benign brain tumors include meningiomas, acoustic neuromas, and pituitary adenomas. While these tumors are not usually life-threatening, they can still cause symptoms depending on their size and location. As they grow, benign tumors can exert pressure on the brain, leading to neurological symptoms such as headaches, seizures, changes in vision, and difficulties with coordination or balance. Treatment for benign brain tumors may involve observation, especially if the tumor is small and does not cause significant symptoms. In some cases, surgical removal of the tumor may be recommended to relieve symptoms or reduce the risk of complications. Depending on the tumor's location and characteristics, other treatment options such as radiation therapy or stereotactic radiosurgery may be considered. Malignant brain tumors, also referred to as brain cancer, are cancerous growths that can invade nearby brain tissue and have the potential to spread to other parts of the central nervous system. Examples of malignant brain tumors include glioblastomas, astrocytoma’s, and medulloblastomas. These tumors can be life-threatening due to their aggressive growth and infiltrative nature. Malignant brain tumors often cause symptoms that progressively worsen over time, including persistent headaches, seizures, cognitive impairments, changes in behavior or personality, and motor deficits. The symptoms can vary depending on the tumor's location within the brain and the extent of its growth. Treatment for malignant brain tumors typically involves a combination of approaches. Surgery is often performed to remove as much of the tumor as possible, followed by radiation therapy to target remaining tumor cells. Chemotherapy may also be used, either orally or delivered directly into the brain (intrathecal chemotherapy). In some cases, targeted therapies or immunotherapy may be considered, depending on the tumor's molecular characteristics.

Survival rates for various diseases, including cancer, can be significantly influenced by early detection and timely access to appropriate medical care. When a disease is detected in its early stages, treatment can be initiated at a point when the disease burden is lower, and the chances of successful intervention are higher. Early detection allows for prompt medical intervention, which can lead to better treatment outcomes and increased chances of survival. It enables healthcare professionals to implement targeted therapies and interventions that are more effective in controlling disease progression and preventing complications. The detection and accurate segmentation of brain tumors at an early stage is crucial for improving survival rates and providing proper medical care. Medical experts face the challenging task of automatically and accurately identifying, classifying, and segmenting brain tumors, as even a small error or misjudgment can have life-threatening consequences for patients. This has prompted us in the field of medical imaging to focus on developing computerized methods for tumor recognition and segmentation.

In this work, we have developed an automatic and accurate method for the detection and segmentation of brain tumors from MRI images. Unlike previous approaches that employed sequential classification and segmentation, we proposed a simultaneous approach using the two-headed UnetEfficientNet models. This allowed us to perform classification and segmentation tasks concurrently, enhancing the overall efficiency of the process. Additionally, we implemented a post-processing technique utilizing connected component labelling to further improve the accuracy of the segmentation results. By combining these innovative methods, we achieved significant advancements in the automatic detection and segmentation of brain tumors, which have potential implications for improving diagnosis and treatment planning in clinical settings.

The manuscript is structured as follows: In Sect. 2, a comprehensive review of the literature related to brain tumor detection and segmentation is provided, highlighting the existing research gap and the contributions of this work. Section 3 outlines the materials and methods employed in implementing the proposed approach, including the methodology, dataset utilized, and the architectural details. The results of the experiments conducted are presented and discussed in detail in Sect. 4. Finally, Sect.  5 offers concluding remarks on the findings and discusses the future scope of the research.

## Literature review

This section focuses on the utilization of machine learning and deep learning models in computer vision for the detection and segmentation of brain tumors in biomedical imaging over the last five years, from 2018 to 2023. The rapid advancement of computer vision techniques has enabled significant progress in this field, offering efficient and accurate solutions for tumor detection and segmentation tasks in medical imaging.

The study by Al-Dmour and Al-Ani (2018) utilized SA (Statistical Analysis) and pixel intensity features with the CFNN (Convolutional Fuzzy Neural Network) model. The proposed approach achieved an impressive accuracy of 98.10% for brain tumor classification on the IBSR18 and IBSR20 datasets. The combination of SA and pixel intensity features proved effective in accurately distinguishing between tumor classes. Jeevakala and Brintha Therese (2018) presented an image enhancement method for MR images using LP (Laplacian pyramid) and SVD (singular value decomposition). The method aimed to improve the perceptibility and segmentation of MR images by sharpening the images and reducing noise. Performance metrics such as AMBE, MSSIM, and PSNR were used to evaluate the effectiveness of the proposed method. The study focused on enhancing the quality of MR images to facilitate accurate diagnosis and segmentation. Devkota et al. (2018) proposed an MMR (Mathematical Morphological Reconstruction) approach for the early detection and diagnosis of brain tumors. The preprocessing stage involved denoising and artifact removal from MR images, followed by segmentation. The tumor classification was performed based on statistical and texture-based features, categorizing tumors as benign or malignant. The study aimed to improve the accuracy of brain tumor diagnosis using the proposed approach.

Talo et al. (2019) employed a CNN (Convolutional Neural Network) model for brain tumor classification using a dataset of 1074 MRI images. The proposed model achieved a high accuracy of 95.23%. The CNN architecture demonstrated its capability in capturing relevant features from the MRI images and effectively classifying brain tumors. Özyurt et al. (2019) introduced a hybrid technique called Neutrosophy-CNN (NS-CNN) for brain tumor classification. The approach involved segmentation of the images, feature extraction using a CNN classifier, and classification using SVM and KNN classifiers. The study utilized a dataset of 160 brain MRI images, consisting of 80 benign and 80 malignant tumors. Performance evaluation with fivefold cross-validation demonstrated the improved performance of the SVM classifier with CNN features, achieving a 95.62% accuracy rate on the validation data. Gupta et al. (2019) proposed an approach that combined GLCM (Gray-Level Co-occurrence Matrix), LBP (Local Binary Patterns), GLRLM (Gray-Level Run Length Matrix), and PHOG (Pyramid Histogram of Oriented Gradients) features with an EC (Ensemble Classifier) model. This combination achieved an accuracy of 97.76% on the BRATS and JMCD datasets. The integration of multiple texture and shape features with the EC model showcased promising results in brain tumor classification. Yang et al. (2019) presented SK-TPCNN (small-kernels two-path CNN) for automatic segmentation of brain MR images, using a random forest classifier. The method involved feature extraction using SK-TPCNN and applying these features to the random forest classifier for optimization. The study aimed to improve the accuracy of brain tumor segmentation by utilizing the proposed architecture. Kalaiselvi et al. (2019) proposed a technique for creating a color (RGB) image by merging color channels formed from multiplexed or multimodal MR images. They performed segmentation and extraction of brain tumors from MR images, specifically using the BRATS dataset. The study aimed to enhance the visualization and identification of brain tumors using a novel color merging technique.

Daimary et al. (2020) presented novel hybrid deep neural models, namely Seg-UNet, U-SegNet, and Res-SegNet, for segmenting brain tumors using the BraTS dataset. These models combined different CNN architectures such as SegNet, U-Net, and ResNet to enhance the accuracy of tumor segmentation. The hybrid models outperformed individual CNN networks, demonstrating their efficacy in segmenting brain tumors.Çinar and Yildirim ( 2020) introduced a Hybrid CNN model for brain tumor classification. This approach achieved an accuracy of 97.20% on a dataset of 253 MRI images. The Hybrid CNN architecture leveraged both convolutional and fully connected layers, effectively capturing spatial dependencies and learning discriminative features for accurate tumor classification.

Viji et al. (2020) proposed a method for classifying brain MRI images using the K-nearest neighbor classifier (KNNC) along with segmentation techniques. They applied preprocessing steps, feature extraction, and a genetic algorithm for optimized feature selection. The KNN classifier was utilized to classify brain MRI images into normal and abnormal categories. Safdar et al. (2020) utilized the YOLO v3 (You Only Look Once) model for brain tumor classification and achieved an accuracy of 96.00% on a dataset of 1961 MRI images. The YOLO v3 model, known for its object detection capabilities, showcased its potential in accurately identifying and classifying brain tumors. Virupakshappa and Amarapur (2020) proposed an AANN + WOA (Artificial Neural Network with Whale Optimization Algorithm) model that combined MWD (Multiscale Wavelet Descriptor), GLCM, GT (Gradient Texture), and moment invariant features. This approach achieved an accuracy of 98.00% on the BRATS 2015 dataset. The integration of multiple features with the AANN + WOA model showcased its effectiveness in accurate tumor classification. Ghassemi et al. (2020) presented a GAN (Generative Adversarial Network) + CNN model for brain tumor classification, achieving an accuracy of 95.60% on a dataset of 3064 MRI images. The GAN + CNN combination allowed for more robust feature extraction and classification, resulting in accurate tumor classification. Naser et al. (2020) developed a classifier using the U-net architecture and a pre-trained VGG-16 CNN model with transfer learning for grading brain tumors. They performed segmentation and grading of tumors using various MRI images, including T1-precontrast, FLAIR, and T1-postcontrast. The CNN model demonstrated effective classification, automatic segmentation, detection, and grading of brain MRI images. Brunese et al. (2020) utilized radiomic features such as FOF (First Order Features), Shape features, GLCM, GLRLM, and GLSZM (Gray-Level Size Zone Matrix) with an EC model. This approach achieved an outstanding accuracy of 99.00% on datasets including REMBRANDT, BraTS 2019, and Radiopaedia (111,205 MRI). The integration of radiomic features and the EC model demonstrated its effectiveness in precise tumor classification. Nalepa et al. (2020) proposed an automated system for analyzing dynamic contrast-enhanced MRI (DCE-MRI) of tumors. They introduced a cubic deep learning model to minimize fitting errors and achieved rapid processing times of less than 3 min on a single GPU. Their system showed promising results in the analysis of DCE-MRI for tumor assessment. In the study conducted by Hashemzehi et al. (2020), a CNN-NADE (Convolutional Neural Network—Neural Autoregressive Distribution Estimator) model was employed for brain tumor classification. The model was trained and evaluated on a dataset of 3064 MRI images. The proposed method achieved a classification accuracy of 95.00%. Although the accuracy is relatively moderate compared to some other models, the utilization of CNN-NADE showcases the potential of combining deep learning techniques with generative models for accurate brain tumor classification.

Karayegen and Aksahin (2020) employed a CNN model for brain tumor classification on a dataset of 257 MRI images. The CNN model was trained to learn discriminative features from the input images and make accurate predictions. The study achieved a classification accuracy of 95.70%. Despite the smaller dataset size, the results demonstrate the potential of CNN models in accurately classifying brain tumors and capturing relevant patterns and features.

Yan et al. (2022) pioneered the development of a sophisticated deep learning imaging signature (DLIS) derived from MRI scans, aimed at prognosticating the 1p/19q status in patients afflicted with low-grade glioma (LGG). Through rigorous training on a dataset comprising 330 samples, they subsequently validated the model using 123 internal and 102 public datasets. Impressively, the DLIS yielded a remarkable ROC score of 98.3% alongside an F1 score of 92.5% on the test dataset. Vankdothu and Hameed (2022) focused on GLRLM (Gray-Level Run Length Matrix) features combined with an SVM (Support Vector Machine) model for brain tumor classification using a dataset of 210 CT (Computed Tomography) images. GLRLM features capture the distribution of voxel intensities in different directions and distances. The proposed approach achieved an impressive classification accuracy of 99.24%. This indicates the effectiveness of GLRLM features and the SVM model in accurately classifying brain tumors based on CT imaging. Shetty et al. (2022) utilized the LBP (Local Binary Patterns) technique combined with a CNN model for brain tumor classification. The LBP method captures local texture patterns in the images, while the CNN model learns to extract high-level features and make accurate predictions. The study used a dataset of 1500 MRI images and achieved a classification accuracy of 98.21%. This demonstrates the effectiveness of LBP features and CNN models for accurate brain tumor classification in MRI images. Mahmud et al. (2023) employed a CNN model for brain tumor classification on a relatively large dataset of 3264 MRI images. The CNN model was trained to learn discriminative features and classify the input images into different tumor categories. The study achieved a classification accuracy of 93.30%. While the accuracy could be further improved, the results indicate the potential of CNN models in accurately classifying brain tumors based on MRI data.

Ramtekkar et al. (2023) focused on GLCM (Gray-Level Co-occurrence Matrix) features combined with an OCNN (One-Class Neural Network) model for brain tumor classification. The GLCM features capture the spatial relationships between voxel intensities in the images, while the OCNN model is trained to distinguish between normal and abnormal brain tissues. The study used a dataset of 253 MRI images and achieved a classification accuracy of 98.90%. This highlights the effectiveness of GLCM features and the OCNN model for accurate brain tumor classification based on MRI data. Saeedi et al. (2023) considered shape, intensity, and model-based features combined with a 2D CNN model for brain tumor classification. The shape features capture the geometric properties of the tumors, intensity features represent the voxel intensities in the images, and model-based features extract information from mathematical models applied to the tumor regions. The proposed approach achieved a classification accuracy of 96.47% on a dataset of 3264 MRI images. This demonstrates the potential of incorporating multiple feature types and utilizing a 2D CNN model for accurate brain tumor classification. Archana and Komarasamy (2023) focused on utilizing segmented features combined with a BKNN (Binary Kernel Neural Network) model for brain tumor classification. The segmented features are derived from the segmented tumor regions, capturing specific characteristics of the tumors. The BKNN model is trained to classify the segmented features into different tumor classes. The study used a dataset of 3064 MRI images and achieved a classification accuracy of 97.70%. This highlights the effectiveness of segment-based features and the BKNN model in accurately classifying brain tumors based on MRI data. Rasheed et al. (2023) combined GLCM (Gray-Level Co-occurrence Matrix) and statistical features with an SVM (Support Vector Machine) model for brain tumor classification. The GLCM features capture the spatial relationships between voxel intensities, while statistical features include mean, standard deviation, skewness, and kurtosis. The proposed method achieved a classification accuracy of 98.00% on a dataset of 3541 MRI images. This demonstrates the effectiveness of the combined GLCM and statistical features, along with the SVM model, for accurate brain tumor classification. Krishnapriya and Karuna (2023) employed a CNN model based on the VGG-19 architecture for brain tumor classification. The VGG-19 model is a deep convolutional neural network capable of learning intricate features from the input images. The study used a dataset of 305 MRI images and achieved an exceptional classification accuracy of 99.48%. This highlights the effectiveness of deep learning models, specifically the VGG-19 architecture, in accurately classifying brain tumors based on MRI data.

Through an extensive review of the current and previous literature on brain tumor detection, researchers have employed various deep-learning techniques to tackle this challenge. These techniques include convolutional neural networks (CNN), U-Net, long short-term memory (LSTM), recurrent neural networks (RNN), transfer learning, hybrid methods, encoders, and many others. While significant contributions have been made by researchers in MR image analysis using these techniques, there is still room for improvement. One noticeable aspect is that many researchers have focused on either segmentation or classification alone, neglecting the integration of both tasks. Furthermore, among the few studies that have combined segmentation and classification, most have adopted a sequential approach, treating these tasks as separate steps rather than considering them simultaneously. Another aspect that stands out is the limited utilization of post-processing techniques. Only a small number of researchers have explored the potential benefits of post-processing methods to improve the accuracy and quality of the results. This highlights an area where further investigation and development can lead to enhanced performance. Moreover, there are notable differences in the achieved classification accuracy and segmentation metrics, such as the Dice score, among the myriad studies. These differences may be attributed to variations in the datasets used, the choice of deep learning models, the specific techniques employed, and the overall methodology adopted in each study. In summary, while significant advancements have been made in brain tumor detection using deep learning techniques, there are still opportunities for improvement. Integrating both segmentation and classification tasks, adopting simultaneous approaches, and exploring the potential of post-processing techniques could contribute to further advancements in the accuracy and reliability of brain tumor detection systems.

Therefore, to address the aforementioned issues and research gaps identified during the literature survey, this paper proposes a novel approach using the two-headed UnetEfficientNets model for the parallel detection and segmentation of brain tumors from MRI images. The proposed model aims to overcome the limitations observed in previous studies and make significant contributions to the field. The main important contributions of this work are highlighted below:

- *Integration of Segmentation and Classification:* Unlike many existing studies that focus on either segmentation or classification alone, the proposed model combines both tasks in a parallel manner. This integration allows for a more comprehensive analysis of brain tumor images and provides a holistic understanding of the disease.
- *Two-Headed Architecture:* The UnetEfficientNets model incorporates two heads, one for segmentation and one for classification. This architecture enables the model to simultaneously generate accurate segmentations of tumor regions and classify the tumor type, leading to a more comprehensive and informative analysis.
- *Utilization of EfficientNets:* The EfficientNet models are utilized in the encoder section of the proposed model. These models are known for their ability to extract deep and fine features, which are crucial for accurate tumor detection and segmentation.
- *Post-Processing Techniques:* The proposed model incorporates post-processing techniques, such as connected component labeling (CCL), to further enhance the segmentation results. This step helps refine the segmentation masks and improve the overall accuracy of the model.
- *Ensemble Technique:* To validate the performance of the proposed model, an ensemble technique is employed. By combining the outputs of multiple models, the ensemble approach aims to improve the overall segmentation accuracy and robustness of the system.
- *Superior Segmentation and Classification Performance:* The model achieves high segmentation metrics (Dice score: 94.03%, Jaccard Index: 98.67%) and exceptional classification metrics (Accuracy: 99.00%, Precision: 99.40%, Recall: 99.00%). This demonstrates its precise detection and segmentation of brain tumors, contributing to improved diagnosis and treatment planning.

Overall, the proposed two-headed UnetEfficientNets model addresses several research gaps and limitations identified in the literature. It combines segmentation and classification tasks, incorporates advanced deep learning architectures, utilizes post-processing techniques, and employs an ensemble approach for improved performance. These contributions aim to advance the field of brain tumor detection and segmentation and provide valuable insights for accurate diagnosis and treatment planning.

## Materials and methods

**Fig. 2:**
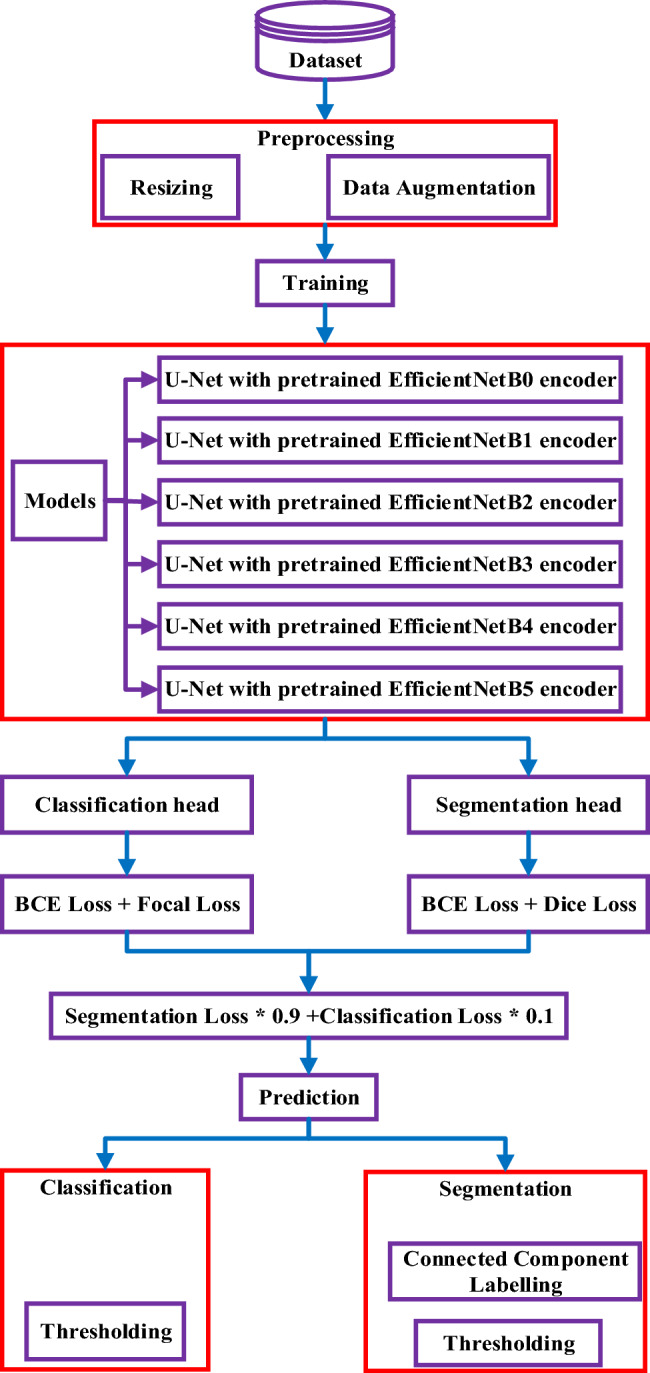
Flow diagrem of two headed UnetEfficientNet model

In this section, we provide a detailed overview of the methods and materials used in our research for the detection and segmentation of brain tumors. Our approach, as depicted in Fig. 2, encompasses several stages and techniques aimed at achieving accurate and efficient tumor detection and segmentation. The first stage of our approach involves the utilization of MRI brain tumor datasets. These datasets consist of MRI images of patients with brain tumors, along with corresponding tumor labels or masks. These datasets serve as the foundation for training and evaluating our model. To prepare the dataset for analysis, we perform preprocessing tasks. One important preprocessing step is resizing the MRI images. This ensures that all images have consistent dimensions, typically achieved by resizing them to a specific width and height. Standardizing the image size facilitates easier data handling and ensures compatibility with the model architecture. Additionally, we employ data augmentation techniques to enhance the dataset. Data augmentation involves applying various transformations to the existing images, such as rotation, scaling, flipping, and cropping. These transformations introduce variations in the dataset, effectively increasing its diversity and enabling the model to generalize better.

Moving on to the model architecture, we leverage the power of Convolutional Neural Networks (CNNs). CNNs have revolutionized computer vision tasks, including tumor detection, due to their ability to automatically learn hierarchical features from images. Several CNN architectures have been developed over the years, ranging from classic models like LeNet-5 and AlexNet to more recent and sophisticated architectures like VGG-16, VGG-19, and ResNet. In our proposed approach, we utilize UnetEfficientNet models with two heads: one for segmentation and the other for classification. The segmentation head employs the Sorensen-Dice coefficient (DICE) as a loss function. DICE measures the agreement between the predicted tumor segmentation and the ground truth tumor mask. The classification head, on the other hand, uses binary cross-entropy loss to classify whether a tumor is present or not. After obtaining the outputs from the segmentation and classification heads, we apply post-processing techniques to refine the results. One such technique is the Connected Component Labeling (CCL) algorithm. CCL helps in accurately delineating tumor regions by labeling connected pixels or regions, providing more precise tumor segmentation.

To evaluate the performance of our proposed model, we employ various performance evaluation metrics. These metrics include IoU (Jaccard Coefficient), DICE coefficient, precision, recall, F1-score, and accuracy. These metrics provide insights into the accuracy, quality, and overall performance of our model in detecting and segmenting brain tumors. By following this comprehensive approach, we aim to achieve accurate and reliable detection and segmentation of brain tumors from MRI images, contributing to improved diagnosis and treatment planning for patients. Throughout the succeeding sections, a detailed analysis and explanation of each stage will be provided, shedding light on the specific techniques and methodologies employed in tumor detection and segmentation from MRI images.

### MRI dataset

The dataset used in this work is a publicly available open-source dataset called the Figshare MRI dataset, which can be downloaded from a source cited as Cheng (2017). This dataset specifically focuses on brain tumors and consists of 2D MRI images along with their corresponding tumor masks and labels. The dataset includes a total of 3064 T1-weighted CE-MRI (Contrast-Enhanced Magnetic Resonance Imaging) images, each with a size of 512 × 512 pixels and a pixel size of 0.49 × 0.49 mm2. In terms of the image properties, each MRI slice has a thickness of 6 mm and a distance of 1 mm between slices. It's worth noting that the dataset covers three different tumor types, although the specific types are not mentioned in this description. To ensure the accuracy of the tumor segmentation, three expert radiologists manually outlined the tumor masks or borders in the MRI images. The dataset consists of three different types of tumors: Meningioma, Glioma, and Pituitary. For Meningioma, there are 82 patients included in the dataset. These patients contribute a total of 708 MRI images.

**Fig. 3:**
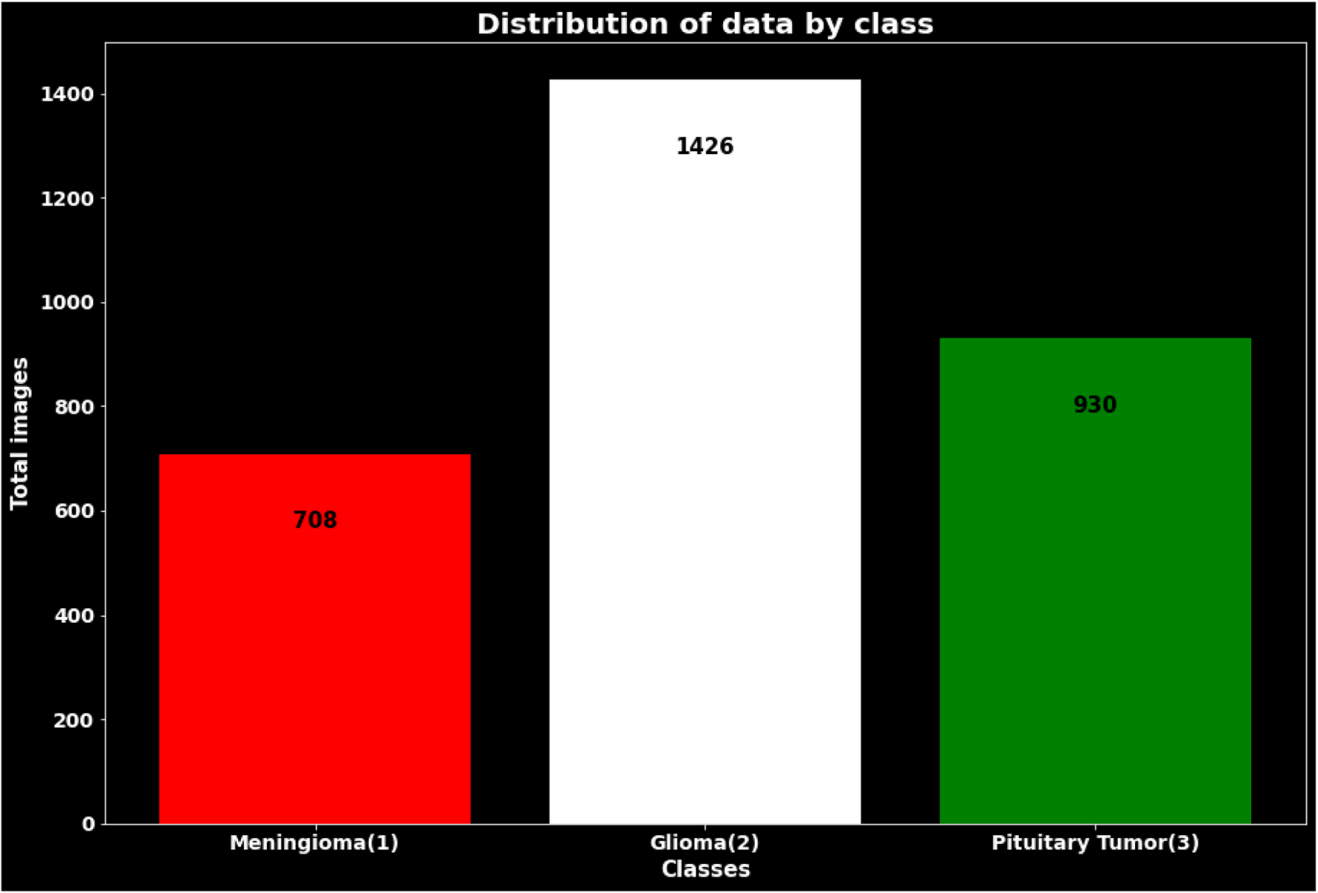
Dataset distribution by types of tumors (Meningioma, Glioma, Pituitary)

The MRI images are further categorized based on the MRI view they represent. Specifically, there are 209 MRI images captured in the axial view, 268 images in the coronal view, and 231 images in the sagittal view. This distribution allows for a comprehensive analysis of the tumor from different perspectives. For Glioma, the dataset includes 89 patients. This group contributes a larger number of MRI images, with a total of 1426. Similar to Meningioma, the MRI images for Glioma are categorized by view. There are 494 images in the axial view, 437 images in the coronal view, and 495 images in the sagittal view. This extensive collection of MRI images enables a detailed examination of Glioma tumors from various angles. The third tumor type in the dataset is Pituitary. It includes 62 patients, providing a total of 930 MRI images. As with the other tumor types, the MRI images for Pituitary tumors are divided into different views. There are 291 images in the axial view, 319 images in the coronal view, and 320 images in the sagittal view. This distribution allows for a comprehensive analysis of Pituitary tumors in different orientations. This diverse and extensive dataset provides a valuable resource for studying and evaluating various aspects of tumor analysis using MRI imaging techniques. Figure 3 presents the distribution of the dataset based on the types of tumors, including Meningioma, Glioma, and Pituitary. The dataset consists of MRI images, and the distribution is shown before any data augmentation. The number of images in each tumor class is depicted, highlighting the varying proportions of the dataset for different tumor types.

### Data pre-processing

**Fig. 4:**
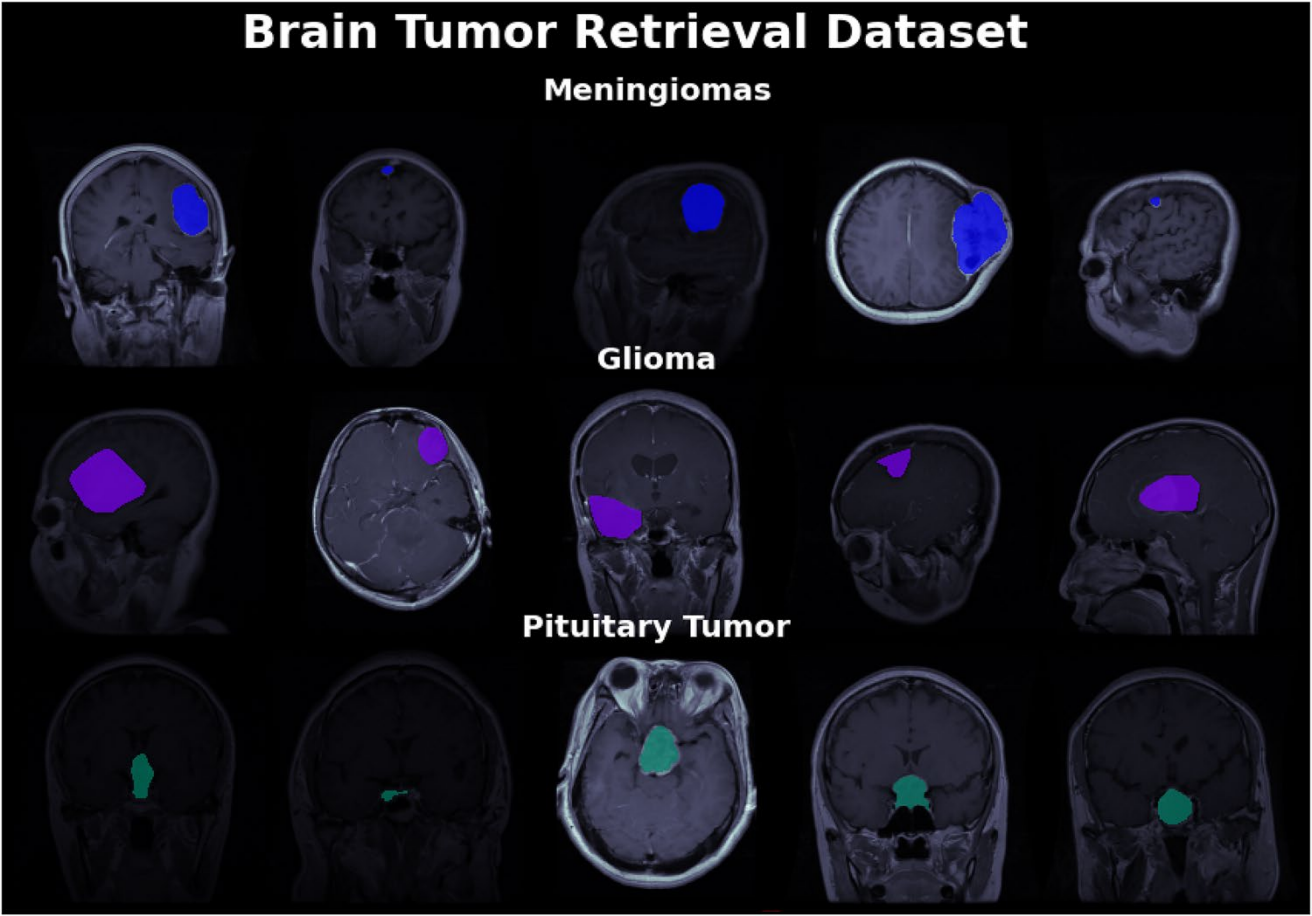
MR images of all tumor classes along with their tumor mask

Pre-processing is a critical step in data analysis that involves preparing the dataset by extracting relevant and meaningful information while removing unwanted or irrelevant data. In computer vision, including the analysis of medical images, preprocessing plays a vital role in ensuring the accuracy and effectiveness of subsequent algorithms or classifiers. In the context of this work, we have focused on the pre-processing of a database used for experimenting with a deep learning model. The database was highly heterogeneous, meaning it contained images with varying sizes and dimensions. To enable efficient segmentation and analysis, it was necessary to standardize the dataset by resizing the images to have the same width and height. This ensured that all images were of a consistent size, facilitating easier comparison and analysis. By standardizing the size of the images, the researchers aimed to overcome the challenges posed by the heterogeneity of the dataset. Inaccurate data or variations in image sizes can hinder the performance of classifiers or algorithms, leading to suboptimal results. Therefore, ensuring efficient segmentation required the dataset to be pre-processed by resizing the images. In this specific work, we have focused on three preprocessing techniques: resizing the MRI images, global pixel normalization, and augmentation. These techniques were applied before performing image segmentation. Figure 4 provides a visual representation of MR images depicting different tumor classes, namely Meningioma, Glioma, and Pituitary, along with their corresponding tumor masks. The images showcase the distinctive characteristics and locations of these tumors within the brain. The tumor masks, highlighted in red, delineate the specific regions that have been segmented as tumor areas in the images.

#### Resizing

Resizing the images involves adjusting their dimensions while maintaining the aspect ratio. In this case, the images are resized to a specific shape, such as 256 × 256 pixels. This means that all the images will have the same width and height of 256 pixels, regardless of their original dimensions. By resizing the images, we achieve consistency in the dataset. This allows for easier processing and analysis, as the images are in a uniform format. Resized images with the same dimensions also facilitate the application of subsequent preprocessing steps and the training of machine learning models. Overall, the resizing step in the preprocessing pipeline ensures that the MRI images are standardized and prepared for further analysis, enabling more accurate and reliable results in brain tumor detection and segmentation tasks. Here are the steps for applying the resizing operation to the MRI images:

- Define the target size for resizing (e.g., 256 × 256 pixels).
- Iterate through each tumor type in the dataset (Meningioma, Glioma, Pituitary).
- For each tumor type, iterate through each MRI view (Axial, Coronal, Sagittal).
- Get the number of MRI images for the current tumor type and MRI view.
- Iterate through each MRI image for the current tumor type and MRI view.
- Load the MRI image.
- Resize the image to the target size using an appropriate resizing function or library.
- Save the resized image to a new location or overwrite the original image.
- Repeat steps 5–8 for all MRI images of the current tumor type and MRI view.
- Repeat steps 3–9 for all MRI views of the current tumor type.
- Repeat steps 2–10 for all tumor types in the dataset.

#### Global pixel normalization

In the second step of the preprocessing pipeline, a technique called global pixel normalization is applied to the cropped images. This step aims to normalize the pixel values of the images to a common scale. The pixel values in the original images typically range from 0 to 255, representing the intensity or brightness of each pixel. However, for better training of deep learning models, it is often recommended to normalize these pixel values to a range between 0 and 1. Normalization involves transforming the pixel values while preserving the relative differences between them. The main idea behind normalization is to bring all the pixel values to a common scale, which helps in preventing any single pixel value from dominating the training process. By normalizing the pixel values between 0 and 1, the images are standardized and brought to a consistent range, making it easier for the deep learning model to learn and extract meaningful features from the data. It is important to note that normalization does not change the relative order or distribution of the pixel values. It only scales the values so that they fall within a specific range, in this case, 0 to 1. Overall, the goal of global pixel normalization in the preprocessing step is to ensure that the pixel values of the cropped images are on a standardized scale, which can improve the training and performance of the deep learning model in subsequent stages of the analysis. The algorithm 1 used for global pixel normalization is presented below.

**Algorithm 1:**
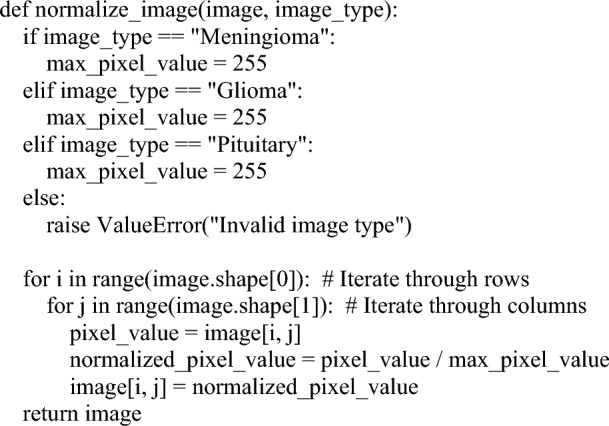
Global pixel normalization

#### Data augmentation

To ensure the effective deployment and learning of Deep Learning (DL) models, it is crucial to train them on high-quality and ample data. Training DL models on large datasets allows them to learn in a more optimal manner and helps prevent issues like overfitting, where the model becomes overly specialized to the training data and performs poorly on new data (Sajjad et al. 2019). Plentiful and high-quality training data significantly enhances the performance of DL models and improves their learning accuracy. Data Augmentation (DA) is an artificial technique used to generate and create variations in the original dataset. It involves applying modifications or changes to the dataset to create a manifold reproduction of the original images using different methods. DA can be performed through data oversampling or data wrapping. Data oversampling involves creating artificial instances and combining them with the existing dataset. It includes techniques such as data fraternization, feature space DA, and the use of Generative Adversarial Networks (GANs) (Shorten and Khoshgoftaar 2019). There are numerous ways to perform data augmentation, including flipping, cropping, resizing, rotation, brightness adjustments, blurring, and more. In this work, 12 types of augmentation techniques were utilized, each with specific parameters, resulting in the creation of 12 replicas of each image (Table 1).

**Table 1:** Dataset description Including tumor types, number of patients, MRI views, and total MRI images

Tumor Type	Number of Patients	Number of MRI Images	MRI view	Number of MRI Images
Meningioma	82	708	Axial	209
			Coronal	268
			Sagittal	231
Glioma	89	1426	Axial	494
			Coronal	437
			Sagittal	495
Pituitary	62	930	Axial	291
			Coronal	319
			Sagittal	320
Total	233	3064	Total	3064

The types of augmentations and their corresponding parameters are outlined in Table 2. The augmentation techniques serve to introduce variations and distortions into the original images, expanding the dataset with diverse examples. By exposing the DL model to these augmented samples, it becomes more robust to different variations and better able to generalize its predictions to unseen data.

**Table 2:** Type of data augmentation and its parameters

S.No	Type of augmentation	Parameters
1	Resize	156
2	Random crop	128
3	Horizontal flip	− 45
4	Flip and rotation (Angle in degree)	45
5	Random rotate	90
6	Shift scale rotate	1.0
7	Transpose	2.0
8	Blur	0.75
9	Gaussian blur(Sigma value)	0.25
10	Random gamma	0.50
11	Random brightness	1.0
12	Normalize	0.25

Data augmentation is a technique applied to the dataset after it has been split into training, validation, and test sets. The purpose of data augmentation is to increase the size and diversity of the training set, which can help improve the model's ability to generalize and perform well on unseen data. The number of images before and after train test split along with % distribution is listed in Table 3. In the context of this study, the dataset was divided into the train, validation, and test sets, with the training set initially accounting for 81% of the data. However, the small size of the original dataset might have limited the model's capacity to learn complex patterns effectively. To address this limitation, data augmentation was applied, resulting in a significant increase in the number of images in the training set. The augmented training set expanded to 29,782 images, representing 87% of the total dataset. This augmentation process introduced more diverse variations of the original images, such as rotations, flips, and zooms, effectively enriching the training data. The validation set also saw an increase in size, with 4,044 images, which accounted for 12% of the dataset. A larger validation set allows for more accurate assessment of the model's performance during training and helps estimate its ability to generalize to new and unseen data. By evaluating the model on a larger validation set, it becomes possible to obtain more reliable insights into its performance and potential strengths and weaknesses. Although the size of the test set remained the same, with 245 images, it serves as an essential component for evaluating the model's performance on completely unseen data. The test set provides an opportunity to assess how well the model generalizes to new samples and how effectively it can detect and segment brain tumors in real-world scenarios.

**Table 3:** Train test split before and after data augmentation

Train test Split (Before Augmentation)					Train test Split (After Augmentation)			
Tumor Type	MRI Images	Train	validation	Test	Train	Validation	Test	Total
Meningioma	708	573	78	57	6882	935	57	7874
Glioma	1426	1155	157	114	13,861	1882	114	15,857
Pituitary	930	753	102	74	9040	1228	74	10,342
Total	3064	2482	337	245	29,782	4044	245	34,071
Distribution (%)	100%	81%	11%	8%	87%	12%	1%	100%

The increase in the percentage distribution of the training and validation sets after data augmentation has a significant impact on the model's performance. With a larger and more diverse training set, the model has a better opportunity to learn intricate patterns and generalize effectively. The augmented dataset provides a more comprehensive representation of the variability in brain tumor images, enhancing the model's ability to accurately detect and segment tumors. In conclusion, the application of data augmentation has positively impacted the training process by expanding the size and diversity of the training set. This, in turn, is expected to improve the UnetEfficientNet model's performance in accurately detecting and segmenting brain tumors from MRI images. The increased distribution of images in the training and validation sets allows the model to learn more effectively, leading to more reliable and robust results.

### Binary cross-entropy loss

Binary cross-entropy (BCE) loss is the summation of sigmoid (σ) activation and cross-entropy (CE) loss, that’s why it is also known as Sigmoid Cross-Entropy (SCE) loss. Opposite to the SoftMax, the BCE loss is independent for each vector class, assuming that the loss for each class in the CNN output model is not affected by any other class values. This means that the assumption of any component belonging to a particular class does not affect the outcome or decision of another class, which is why it is used for multi-label classification (Gómez 2018). The CE loss for an individual example is given by the equation (Sadowski 2020):

1 $$CE=-\sum_{i=1}^{C}{t}_{i}{{\text{log}}(f(P}_{i}))+(1-{t}_{i}{){\text{log}}(1-f(P}_{i}))$$

2 $${f(P}_{i})=\frac{1}{1+{e}^{{P}_{i}}}$$

3 $${P}_{i}=\sum_{j=1}{h}_{j}{w}_{ij}$$

where, in Eqs. (1), (2), and (3), ‘C’ is the number of classes, ‘t’ is target vector, h is the hidden layer and ‘w’ is the weight vector. The value of C decides the number of classes to be predicted, for example, C=2 is set for binary classification. In the case of a binary problem, the loss is calculated for the individual class and then summed to calculate the overall loss. The gradient of the binary problems is also computed using backpropagation methods. To deal with the two class binary problems, P_1_ and t_1_ represent the score or prediction and ground truth or target value for class C_1_, similarly P_2_ = 1−P_1_ and t_2_ = 1−t_1_, represent the prediction and target, respectively for class C_2_.


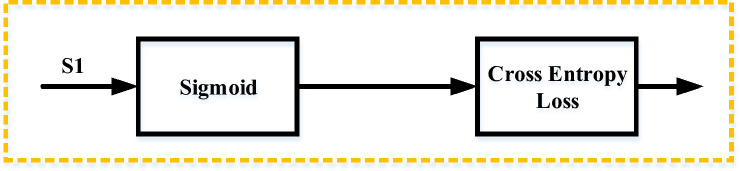


Hence, the cross entropy (CE) loss can also be given by Eq. (4):

4 $$CE=\left\{\begin{array}{l}-{{\text{log}}(f(P}_{1}))\, if\, {t}_{1}=1\\ -{{\text{log}}\,(1-f(P}_{1}))\, if\, {t}_{1}=0\end{array}\right.$$

where t_1_ = 0 represents the negative for the given sample and t_1_ = 1 represents positive for the class C_1_ = C_i_. Here, the activation function mainly depends on the predictions of the class C_1_ = C_i_ instead of other classes in C. The gradient corresponding to each prediction Pi in P will only be influenced by the loss provided by the two-class problem, and equation for the gradient corresponding to Pi = P is given by Eq. (5):

5 $$\frac{\partial }{\partial {P}_{i}}\left(CE\left(f\left({P}_{i}\right)\right)\right)={t}_{1}(f({P}_{1}-1)+{(1-t}_{1}){f(P}_{1})$$

where, the function f() represents the sigmoid activation function and given by the Eq. (6):

6 $$f\left(x\right)=\frac{\partial }{\partial {P}_{i}}\left(CE\left(f\left({P}_{i}\right)\right)\right)=\left\{\begin{array}{l}f\left({P}_{i}\right)-1,\, if \,{t}_{i}=1\\ {f(P}_{i}), \,if \,{t}_{i}=0\end{array}\right.$$

### Focal loss (FL)

It was mainly introduced to solve the detection of one-stage situation for highly imbalanced scenario between background and foreground by Lin et al. (2020) and they achieved better accuracy using RetinaNet model with focal loss. It is a kind of cross-entropy loss that measures the loss of each sample based on the classification error. The reason behind this is that if a data or image is already accurately classified by the CNN model, it does not contribute much to the loss, this means that the loss contribution is reduced. Therefore, by applying this approach, the issue of class imbalance between the precursor and the background is resolved, thereby focusing indirectly on the disadvantages in those problematic classes. In addition, the focal loss measures the contribution of each class to a more pronounced class balance. Similar to the BCE, this also uses sigmoid activation and considering the binary CE loss, it can be defined mathematically in Eq. (7):

7 $$FL=-\sum_{i=1}^{C}{(1-{P}_{i})}^{\gamma }{t}_{i}{{\text{log}}(P}_{i})$$

where γ is the focusing parameter and the value (1 − Pi)^γ^ is known as modulating factor. The modulating factor with γ >  = 0 minimizes the effect of samples accurately classified into losses and if γ = 0, the FL becomes is equal to BCE loss. The focal loss is given by the Eq. (8):

8 $$FL=\left\{\begin{array}{c}-{(1-{P}_{1})}^{\gamma }{{\text{log}}(P}_{1}) \,if\, {t}_{1}=1\\ -{(1-\left(1-{P}_{1}\right))}^{\gamma }{{\text{log}}(1-P}_{1})\, if\, {t}_{1}=0\end{array}\right.$$

where we have divided the equation if C_i_ = C_1_ is negative and hence, class C_2_ is positive. In the case of BCE loss, we have P_2_ = 1-P_1_ and t_2_ = 1-t_2_, but in the case of FL it becomes more complex when constituting the loss function due to the modulating factor (1 − Pi)^γ^, but it can be simplified using CE gradient equation. The gradient equation for C_i_ is positive (t_i_ = 1) is given by:

9 $$\frac{\partial y}{\partial {P}_{i}}\left(FL(f({P}_{i}))\right)={\left(1-f\left({P}_{i}\right)\right)}^{\gamma }\left(\gamma f\left({P}_{i}\right){\text{log}}\left(f\left({P}_{i}\right)\right)+f\left({P}_{i}\right)-1\right) \,if \,{t}_{1}=1$$

Here also f() is sigmoid activation function. The gradient equation for C_i_ = is negative (t_i_ = 0) can be obtained by replacing the f(P_i_) by 1-f(P_i_) in the above Eq. (9).

### Image segmentation connected component labelling (CCL)

Image segmentation is a fundamental task in computer vision and plays a crucial role in various applications, including brain tumor detection. It involves dividing an image into meaningful and coherent regions or segments based on certain characteristics, such as color, texture, intensity, or spatial proximity. The goal of image segmentation is to extract and isolate objects or regions of interest within an image. In the context of brain tumor detection, image segmentation is used to identify and delineate the tumor region from the surrounding healthy brain tissue. This process is essential for accurate diagnosis, treatment planning, and monitoring the progression of the tumor. By segmenting the tumor region, medical professionals can obtain valuable insights into its size, shape, location, and other characteristics, aiding in the assessment of tumor malignancy and guiding treatment decisions. There are various segmentation techniques used in brain tumor detection, ranging from traditional methods to advanced deep learning approaches. Traditional methods include thresholding, region-growing, watershed transform, and active contours (snakes). These techniques often rely on predefined rules, heuristics, or mathematical models to distinguish between tumor and non-tumor regions based on intensity or texture properties.

In this work, we have utilized connected component labelling (CCL) algorithm for accurately segmenting the tumor regions. Connected Component Labelling (CCL) is a segmentation algorithm commonly used in image processing and computer vision to identify and label individual connected regions or objects within an image. The goal of CCL is to partition an image into distinct regions based on the connectivity of pixels. The CCL algorithm works by examining the pixels in the image and assigning labels to connected components. A connected component is a group of pixels that share the same property or characteristic, such as color or intensity, and are connected in a spatial sense. The CCL algorithm follows a step-by-step process:

- Pixel traversal: The algorithm traverses through each pixel in the image, starting from the top-left corner and moving row by row or using a scanning pattern.
- Pixel connectivity: For each pixel, its connectivity to its neighboring pixels is determined. Typically, a 4-connectivity or 8-connectivity is used, where adjacent pixels are considered connected either vertically, horizontally, or diagonally.
- Label assignment: When a newly connected component is encountered, it is assigned a unique label. The labels are usually integers starting from 1 and incrementing for each new component.
- Label propagation: As the algorithm progresses, the labels are propagated to neighboring connected pixels. If a neighboring pixel has the same property or characteristic, it is assigned the same label. This process continues until all connected components in the image have been labeled.
- Post-processing: Optionally, post-processing steps can be applied to refine the segmentation results. This may include filtering out small or noisy components based on their size or applying additional criteria specific to the application.

The output of the CCL algorithm is an image where each connected component is assigned a unique label. This labeled image can be further analyzed or used for various purposes, such as object recognition, object tracking, or feature extraction. CCL is a versatile and widely used segmentation algorithm due to its simplicity and effectiveness in identifying and labeling connected regions in an image. It is especially useful in applications where objects or regions of interest need to be isolated and analyzed separately from the background or other objects. The utilized CCL technique is presented in Algorithm 2.

Note that this algorithm assumes a binary image with foreground (object) pixels labeled as 1 and background pixels labeled as 0. The algorithm can be modified to handle grayscale or color images by incorporating appropriate connectivity criteria and label assignment methods. The specific implementation details, such as the data structures used and the connectivity definition, may vary depending on the programming language and environment you are working with.

**Algorithm 2:**
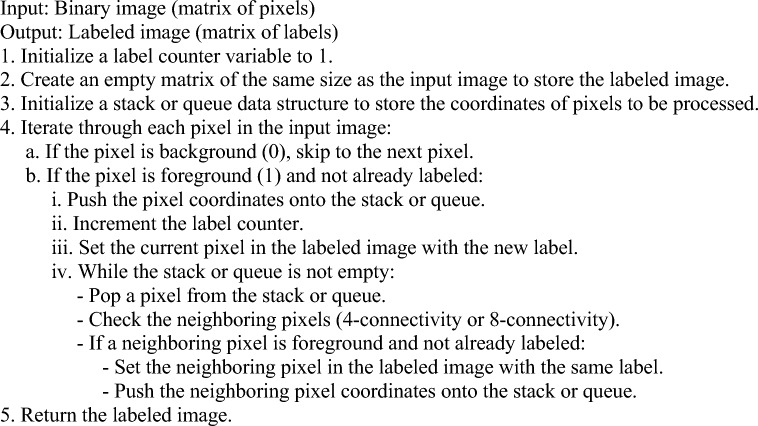
Proposed CCL technique

### Two-headed UnetEfficientNet model

In our work, we have introduced a novel approach called the two-headed UnetEfficientNet model for brain tumor detection and segmentation in MR images. This model is based on Convolutional Neural Networks (CNNs) and incorporates deep learning techniques to achieve both classification and segmentation outcomes for brain tumors. The architecture of the proposed model is designed and modified to efficiently perform both tasks simultaneously. By leveraging the power of CNNs and the Unet architecture, the model can effectively classify whether an image contains a brain tumor or not, as well as accurately segment the tumor regions within the image. The two-headed UnetEfficientNet model offers several advantages. First, it enables a comprehensive analysis of brain tumors by providing both classification and segmentation results, allowing for a more complete understanding of the tumor presence and location. Second, by combining classification and segmentation tasks within a single model, it reduces computational complexity and improves overall efficiency.

CNN, also known as Convolutional Neural Network, has brought about a significant revolution in the field of computer vision. It has emerged as a powerful and efficient model for various visual recognition tasks. Since the introduction of the first CNN model for document verification by Lecun et al. (1998) in 1998, researchers have developed numerous CNN architectures with advancements and modifications. These CNN models include iconic names such as LeNet-5, AlexNet, VGG-16, Inception-v1, Inception-v3, ResNet-50, U-net, Xception, Inception-v4, Inception-ResNets, ResNeXt-50, Unet, and many others. Each of these models offers unique modifications and improvements to the original CNN architecture, enhancing its capabilities in different aspects. CNN is particularly well-suited for automated feature extraction tasks due to its inherent structure. It comprises specialized filters and layers that allow it to detect edges, extract features, and recognize patterns in images. Initially designed for tasks like edge detection, segmentation, and object detection, CNN has expanded its applications to various domains within the machine learning field. The simplicity and versatility of CNN have made it widely applicable in different areas of machine learning. It has proven to be successful in computer vision tasks, text recognition, data mining, speech and signal processing, image and object detection, and numerous other domains. CNN's ability to extract relevant features from raw data without explicit human intervention has made it a preferred choice for many visual recognition tasks.

Unet Among all the CNN models was specially designed for the medical image segmentation task and many modified version is available for different application of segmentation task. The architecture of U-Net is inspired by the shape of the letter "U," which is reflected in its overall structure. The U-Net architecture consists of two main parts: the contracting path (Encoder) and the expansive path (Decoder). The contracting path is similar to the encoder part of a typical CNN, which is responsible for capturing the context and extracting high-level features from the input image. It consists of multiple layers of convolutional and pooling operations, which progressively reduce the spatial dimensions of the input while increasing the number of feature channels. The expansive path, on the other hand, is the decoder part of the network. It is responsible for upsampling the feature maps to the original input size and generating the final segmentation map. The expansive path consists of upconvolutional layers, also known as transposed convolutions or deconvolutions, which increase the spatial resolution of the feature maps. These layers are followed by concatenation with the corresponding feature maps from the contracting path, allowing the network to combine both low-level and high-level features. To improve the localization accuracy of the segmentation, skip connections are introduced between the contracting and expansive paths. These skip connections directly connect feature maps from the contracting path to the corresponding layers in the expansive path. This allows the network to access both local and global context information during the upsampling process, which helps in preserving spatial details and improving segmentation accuracy.

Similarly, one of the most efficient DL models which is utilized for deep feature extraction and classification task is EfficientNet model. The EfficientNet architecture introduces a compound scaling method that uniformly scales the network's depth, width, and resolution. This is achieved by applying scaling coefficients to each dimension. The depth scaling coefficient (φ_d_) determines the number of layers in the network. The depth scaling is calculated by the Eq. (10), where alpha is a constant that controls the network growth rate and increasing φ_d_ results in a deeper network with more layers.

10 $${\varphi }_{d={\alpha }^{\varphi }}$$

Similarly, the width scaling coefficient (φ_w_), determines the number of channels in each layer. It is calculated by the Eq. (11), where beta is a constant that controls the network width multiplier and increasing φ_w_ leads to a wider network with more channels in each layer.

11 $${\varphi }_{w={\beta }^{\varphi }}$$

The resolution scaling coefficient, (φ_r_), determines the input image resolution. It is calculated by Eq. (12), where gamma is a constant that controls the network resolution multiplier and increasing φ_r_ results in higher-resolution input images.

12 $${\varphi }_{r={\gamma }^{\varphi }}$$

The compound scaling method combines these scaling coefficients to determine the network architecture. Given an initial base network architecture, the depth, width, and resolution of each layer are scaled according to the corresponding scaling coefficients. Mathematically, the number of layers L, number of channels C, and input image resolution H x W in the EfficientNet architecture can be expressed by the Eqs. (13), (14), (15), and (16), respectively.

13 $$L = round({\varphi }_{d} * d)$$

14 $$C= round({\varphi }_{w} * w)$$

15 $$H = round({\varphi }_{r} * h)$$

16 $$W = round({\varphi }_{r} * w)$$

Here, d, w, h, and w are the initial values for the base network architecture. The round() function ensures that the resulting values are integers.

EfficientNet models offer a range of variants, consisting of EfficientNet-B0 to EfficientNet-B7. While each variant introduces architectural variations, they all incorporate common blocks or operators. Table 4 provides detailed architectural information for EfficientNet-B0 to EfficientNet-B5 models, including the common blocks, layer numbers (L_i_), number of channels (C_i_), total number of layers, and total parameters. This tabulated data allow for a comprehensive understanding of the architectural characteristics and scalability across the different EfficientNet models.

**Table 4:** Architecture details of EfficientNet-B0 to EfficientNet-B5 models

Stage (i)	Operator	B0		B1		B2		B3		B4		B5	
		C_i_	L_i_	C_i_	L_i_	C_i_	L_i_	C_i_	L_i_	C_i_	L_i_	C_i_	L_i_
1	Conv3 × 3	32	1	32	1	32	1	40	1	48	1	48	1
2	MBConv1, k3 × 3	16	1	16	2	16	2	24	2	24	2	24	3
3	MBConv6, k3 × 3	24	2	24	3	24	3	32	3	32	4	40	6
4	MBConv6, k5 × 5	40	2	40	3	48	3	48	3	56	4	64	6
5	MBConv6, k3 × 3	80	3	80	4	88	4	96	5	112	6	128	8
6	MBConv6, k5 × 5	112	3	112	4	120	4	136	5	160	6	176	8
7	MBConv6, k5 × 5	192	4	192	5	208	5	232	6	272	8	304	10
8	MBConv6, k3 × 3	320	1	320	2	352	2	384	2	448	2	512	3
9	Conv1 × 1 & Pooling & FC	1280	1	1280	1	1408	1	1536	1	1792	1	2048	1
Total No. of Layers		241		339		339		384		474		576	
Total Parameters		5.3 M		7.8 M		9.2 M		12 M		19 M		30 M	

**Fig. 5:**
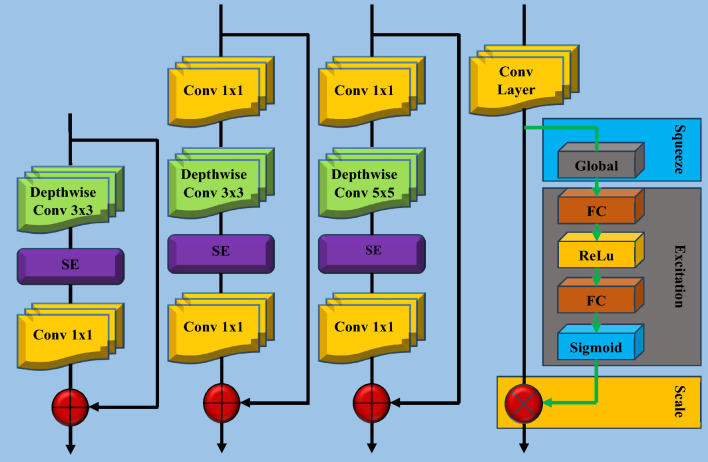
Architectural illustration of EfficientNet-B0 components: (**a**) MBConv1, (**b**) MBConv6 (3 × 3), (**c**) MBConv6 (5 × 5), and (**d**) Squeeze and Excitation (SE) Block

**Fig. 6:**
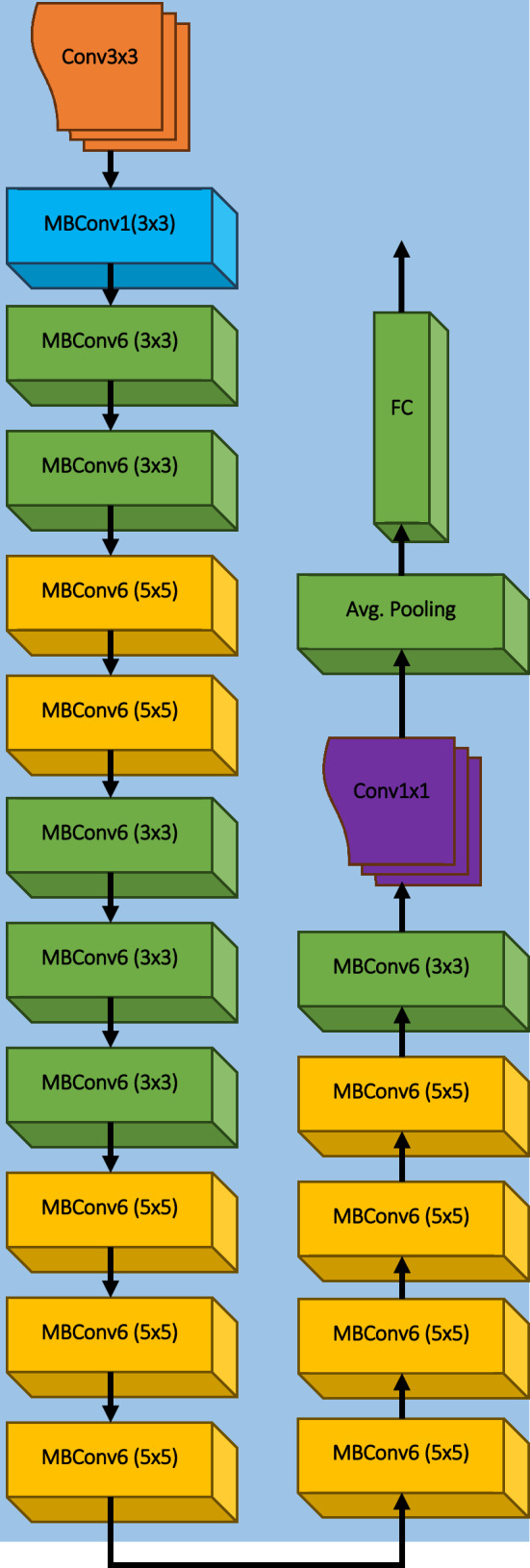
Architecture of the EfficientNet-B0 model

The EfficientNet models utilize several common operators or blocks that are fundamental to their architecture. These include MBConv1, MBConv6 (3 × 3), MBConv6 (5 × 5), and the Squeeze and Excitation (SE) Block. The MBConv blocks, specifically MBConv1 and MBConv6, refer to the MobileNetV2 Inverted Residual Blocks with different kernel sizes. The architectural diagrams of these operators can be found in Fig. 5, providing a visual representation of their structure. Additionally, EfficientNet-B0 serves as the base model for all other variants. Figure 6 specifically illustrates the architectural design of EfficientNet-B0, showcasing its building blocks. It is important to note that all other EfficientNet models, such as B1 to B5, also adopt the same building blocks as EfficientNet-B0. However, they differ in terms of the number of layers, channels, parameters, and other configuration details. These variations are outlined in Table 4, providing a comprehensive comparison of the architectural differences between the different EfficientNet models. For the specific work, six variants of the EfficientNet models, ranging from B0 to B5, have been employed.

**Fig. 7:**
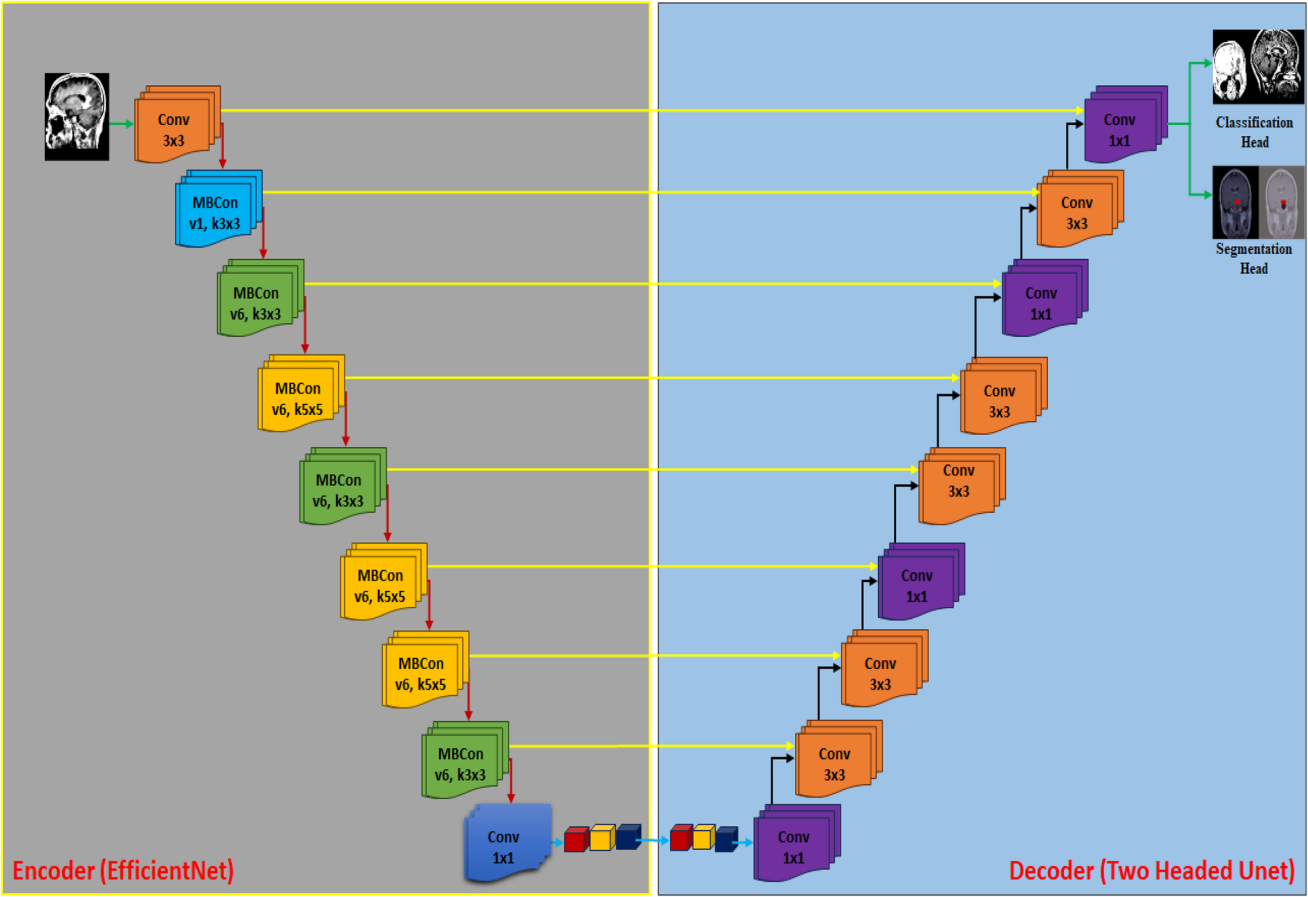
Proposed two-headed UnetEfficientNet model architecture

We have introduced a new DL model called UnetEfficientNet by combining the modified Unet architecture with the EfficientNets models. The main motivation behind this combination is to leverage the deep and fine features extraction capabilities of EfficientNets in the encoder section of the UnetEfficientNet model. Figure 7 illustrates the complete architecture of the proposed two-headed UnetEfficientNet model, which consists of a classification head and a segmentation head. In the encoder section (also known as the contracting path), the building blocks of EfficientNets are utilized. The input MRI image is resized to 256 × 256x3 and fed into the encoder section to extract deep features. The encoder section includes various building blocks such as Conv (3 × 3) for performing convolution operations on the input images. Additionally, MBConv1 (k3 × 3), MBConv6 (k3 × 3), MBConv6 (k5 × 5), Conv1 × 1, Pooling, and Fully connected layers (FC) are employed. In the Inverted Residual Block, Depth Wise Convolution operations along with Squeeze and Excitation (SE) blocks are applied.

These blocks optimize computational efficiency by reducing the number of parameters while maintaining the model's capacity and representational power. Where, Conv (3 × 3) block performs convolution operations on the input images using a 3 × 3 kernel. It helps in capturing local features and patterns. MBConv1 (k3 × 3) block represents the MobileNetV2 Convolutional Block with a 3 × 3 kernel. It applies depthwise separable convolutions, reducing computational complexity while preserving information. MBConv6 (k3 × 3) block is an extension of MBConv1, where the depthwise separable convolution is repeated six times. It allows for capturing more complex and abstract features from the input. Similar to MBConv6 (k3 × 3), this block repeats the depthwise separable convolution operation six times but with a 5 × 5 kernel. It helps in capturing larger receptive fields and more spatial context. Conv1 × 1 block performs 1 × 1 convolutions, which are used for dimensionality reduction and feature compression. It reduces the number of channels and computational load in subsequent layers. Pooling operations, such as max pooling or average pooling, are applied to downsample the feature maps and capture the most salient information. Fully connected layers (FC) layers are responsible for capturing global information and mapping the extracted features to the desired output space. In addition to these building blocks, the Inverted Residual Block, which incorporates depthwise separable convolutions and Squeeze and Excitation (SE) blocks, is utilized to optimize computational efficiency. The depthwise separable convolutions reduce the number of computations, while the SE blocks help in recalibrating the channel-wise feature responses.

In the proposed UnetEfficientNet model, the skip connections are utilized to connect the feature maps from the EfficientNet-based encoder (contracting path) to the corresponding layers in the modified Unet-based decoder (expanding path). This architecture allows the model to effectively combine features from different scales, resulting in a more accurate and detailed segmentation of brain tumors. The skip connections play a critical role in preserving spatial details and leveraging both local and global context information. They allow the UnetEfficientNet model to combine features from different scales, enabling it to capture both fine-grained details and holistic understanding of the brain tumor regions. This comprehensive integration of features helps enhance the model's segmentation accuracy and enables it to effectively delineate the tumor boundaries. The skip connections play a critical role in preserving spatial details and leveraging both local and global context information. They allow the UnetEfficientNet model to combine features from different scales, enabling it to capture both fine-grained details and holistic understanding of the brain tumor regions. This comprehensive integration of features helps enhance the model's segmentation accuracy and enables it to effectively delineate the tumor boundaries.

In our UnetEfficientNet model, the expanding path (decoder) is a modified version of the Unet architecture and incorporates two-headed outputs for classification and segmentation tasks. The decoder utilizes Upconvolutional layers, which are responsible for increasing the spatial resolution of the feature maps and upsampling them to match the original input size. These layers play a crucial role in reconstructing high-resolution segmentation maps from the encoded feature representations. To improve segmentation performance, skip connections are employed in our model. These connections allow the decoder to access feature maps from the corresponding layers in the EfficientNet-based encoder section. By combining the feature maps from both the encoder and decoder paths, our model can leverage both low-level and high-level feature representations, enabling precise localization and refinement of the tumor segmentation.

The skip connections are achieved by concatenating the feature maps from the encoder section with the upsampled feature maps in the decoder. This concatenation operation enables our model to incorporate detailed information from the encoder section, helping in accurate localization and fine-grained segmentation of the tumor regions. Additionally, we employ additional convolutional layers after the concatenation step. These layers further refine the feature maps and capture more intricate information. By applying these convolutional layers, our model can extract detailed patterns and structures from the segmented regions, enhancing its ability to produce precise and accurate segmentation results. Overall, by combining the upsampled feature maps, skip connections, and additional convolutional layers, our UnetEfficientNet model achieves effective localization, refinement, and segmentation of brain tumors. The incorporation of skip connections and the concatenation of feature maps enable our model to leverage both local and global information, resulting in accurate and detailed segmentations that can assist in clinical decision-making and treatment planning.

In our specific implementation, we have utilized Conv (3 × 3) and Conv (1 × 1) convolution layers in combination with upconvolution and concatenation operations in the UnetEfficientNet model. The final model consists of two heads, achieved by modifying the last layer, allowing it to provide outputs for both classification and segmentation tasks. The encoder section of our model varies by employing different EfficientNets, specifically EfficientNet-B0 to EfficientNet-B5. The choice of EfficientNet variations enables the extraction of deep and fine-grained features from the input images.

To ensure compatibility between the encoder and decoder sections, we modify the Unet decoder accordingly to match the feature maps of both sections. For classification, we employ the binary cross-entropy loss (BCE) in combination with the focal loss. This combination helps optimize and generalize the classification results, allowing for accurate predictions of tumor presence or absence. In terms of segmentation, we utilize the BCE loss with Dice score to achieve more effective segmentation outcomes. By optimizing both the segmentation loss and classification loss, with a weight distribution of 90% for segmentation and 10% for classification, we aim to obtain improved predictive outputs. Furthermore, we apply post-processing techniques, including Connected Component Labeling (CCL), to refine the classification and segmentation results. This post-processing step aids in improving the accuracy and cohesiveness of the predicted tumor regions, ultimately enhancing the overall performance of our model in both classification and segmentation tasks.

### Performance assessment metrics

In the context of evaluating DL models for detection and segmentation tasks, parameter evaluation metrics (PEM) play a vital role in measuring performance and efficiency.

While classification tasks commonly rely on metrics like accuracy, sensitivity, specificity, precision, AUC, F-Score, and MCC, object detection and segmentation tasks prioritize the use of Jaccard Index or Intersection over Union (IoU). IoU is preferred because it not only evaluates the accuracy of detection but also quantifies the extent of overlap between the predicted and actual segments. For this work mentioned, we have chosen five evaluation metrics to assess the performance of their proposed model. These metrics include IoU (Jaccard Coefficient), Sorensen-Dice coefficient (DICE), Precision, Recall, and accuracy. Each metric serves a unique purpose in evaluating different aspects of the model's performance. These evaluation metrics are utilized to comprehensively evaluate the performance of the proposed model in terms of detection accuracy, segmentation quality, precision, recall, and overall correctness. Table 5 presents the evaluation metrics used to assess the performance of DL models in object detection and segmentation tasks. Each metric is accompanied by its corresponding formula, definition, and application in evaluating different aspects of the model's performance. The metrics include IoU (Jaccard Coefficient), Sorensen-Dice Coefficient (DICE), Precision, Recall, F1-Score, and Accuracy. These metrics provide insights into the accuracy, similarity, precision, recall, and overall correctness of the model's predictions.

**Table 5:** Performance evaluation metrics for DL models in object detection and segmentation

Metric	Formula	Definition	Application
IoU (Jaccard Coefficient)	$IoU =\frac{ Intersection\, Area }{Union \,Area}$ $J\left({\text{A}},\mathrm{ B}\right)=\frac{\left|{\text{A}}\cap {\text{B}}\right|}{\left|{\text{A}}\cup {\text{B}}\right|}$	Measures the overlap between predicted and ground truth masks	Assessing the accuracy and quality of object segmentation
Sorensen-Dice Coefficient (DICE)	$DICE = \frac{2 * Intersection\, Area}{\left(Sum\, of \,Area\, in\, both\, masks\right)}$ $DICE\left(A,B\right)=\frac{2*|{\text{A}}\cap {\text{B}}|}{\left|{\text{A}}\right|+|{\text{B}}|}$	Measures the agreement between predicted and ground truth masks	Evaluating the similarity and accuracy of object segmentation
Precision	$Precision ({\text{Pre}}) =\frac{ TP}{(TP+ FP)}$	Measures the accuracy of positive predictions	Assessing the model's ability to correctly identify positive instances
Recall	$Recall (Rec) =\frac{TP}{TP+FN}$	Measures the sensitivity or true positive rate	Evaluating the model's ability to correctly detect positive instances
Accuracy	$Accuracy(Acc)= \frac{TP+TN}{TP+FN+TN+FP}$	Measures the overall correctness of predictions	Evaluating the overall performance and correctness of the model

#### IoU (Intersection over Union)

**Fig. 8:**
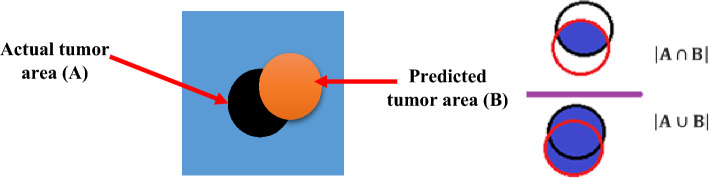
llustration of Intersection over Union (IoU) Calculation

In the case of tumor detection, the IoU (Jaccard index) is used to quantify the overlap or similarity between the predicted tumor area and the actual tumor area (ground truth). It is defined as the ratio of the intersection of the predicted tumor area and the actual tumor area to their union. The IoU score provides a measure of how well the predicted tumor area aligns with the ground truth. To determine whether a prediction is classified as true positive (TP) or false positive (FP), a threshold value is set for the IoU. If the IoU value is greater than the threshold (IoU > threshold), it is considered a true positive. If the threshold value is greater than the IoU (threshold > IoU), it is considered a false positive. In the context of tumor detection, a commonly used threshold value for determining TP and FP is 0.5. If the IoU between the predicted tumor area and the ground truth is greater than 0.5, it is classified as a true positive. Otherwise, it is classified as a false positive. The ground truth (GT) represents the actual tumor area in the image, denoted as A, and the predicted tumor area is represented by B. The IoU score is calculated by taking the intersection of A and B and dividing it by the union of A and B. Figure 8 demonstrates the predicted MRI image with the labeled tumor area, highlighting the overlap between the predicted and actual tumor areas. Using the IoU and the defined threshold, tumor detection algorithms can assess the accuracy and quality of their predictions, distinguishing between true positives and false positives based on the level of overlap between the predicted and actual tumor areas.

## Result and discussion

### Result

In our work, we aimed to develop an effective approach for tumor detection and segmentation using a combination of Unet and EfficientNet models. We utilized the Python programming environment with the PyTorch library to implement our methodology. The hardware setup for our experiments consisted of a system equipped with a 1TB SSD hard drive, 16 GB RAM, NVIDIA GPU, and i7 Pentium core CPU. To begin, we obtained a benchmarked dataset from Figshare, which comprised 3064 MR brain images. These images had varying shapes and sizes, so we applied preprocessing techniques to standardize them. Specifically, we resized all the images to a uniform shape of 256 × 256 × 3 using appropriate image manipulation functions. Additionally, we performed global normalization on the pixel values of the images to ensure consistency and remove any potential biases. Considering the limited size of our dataset, we employed data augmentation techniques during the training phase. This augmentation process involved applying twelve different types of transformations to the training dataset. By introducing variations in the training data, we aimed to enhance the model's generalization ability. However, it's important to note that the test dataset remained unaltered to ensure unbiased evaluation during the model assessment. With the augmented and preprocessed dataset prepared, we proceeded to train our proposed UnetEfficientNet models for tumor detection and segmentation. The architecture of our models featured two heads, combining the strengths of Unet and EfficientNet models. The encoder section of the Unet employed EfficientNet models, which are known for their powerful feature extraction capabilities. On the other hand, the decoder section utilized the Unet model, which excels in capturing fine details and generating precise segmentation masks. By employing this two-headed architecture, our models were able to generate both classification and segmentation outputs. The classification head focused on identifying whether a tumor was present or not in an image, while the segmentation head produced pixel-level masks to precisely delineate the tumor boundaries. This dual functionality allowed us to tackle both tasks simultaneously and leverage the complementary strengths of Unet and EfficientNet models.

We utilized common optimization hyperparameters, outlined in Table 6, for all models except the batch size. These hyperparameters, including the optimizer, learning rate scheduler, segmentation loss, classification loss, and optimization loss, were carefully selected to ensure effective training of the models and accurate performance evaluation. To ensure an unbiased comparison between the UnetEfficientNet-B0 to UnetEfficientNet-B5 models, we have utilized the same number of epochs for each experiment. This approach allows for a fair evaluation of the models' performance by ensuring that they were trained for an equal duration. By training all models for the same number of epochs, we eliminate any potential discrepancies in training time and ensure that each model has had an equal opportunity to converge and learn from the data. By maintaining consistency in the number of epochs across the experiments, we can directly compare the results of the UnetEfficientNet-B0 to UnetEfficientNet-B5 models and assess their relative performance. This enables us to evaluate the impact of architectural variations and determine the most effective model for the given task of brain tumor classification and segmentation. By employing the same number of epochs, we facilitate a more reliable and unbiased comparison between the different UnetEfficientNet models, allowing us to draw meaningful conclusions regarding their performance and effectiveness in the context of our study. To assess the effectiveness of our proposed approach, we employed five models and evaluated their performances. These models were specifically designed to classify and segment tumors into three classes: Meningioma, Glioma, and Pituitary. By leveraging the strengths of Unet and EfficientNet models, our two-headed UnetEfficientNet models showed promising results in both the classification and segmentation tasks for the analysis of brain tumors. Each of these optimization hyperparameters utilizes for our work is explained in details.

**Table 6:** Optimization hyperparameters used for training the models

Model	Batch size	Optimizer	lr scheduler	Segmentation Loss	Classification Loss	Optimization Loss
UnetEfficientnet-B5	2	AdamW—decoder lr: 1e-2 encoder lr: 1e-3	ReduceLROnPlateau—factor: 0.15, patience:2	BCE*0.7 + DICE*0.3	Focal + BCE	Segmentation loss*0.9 + classification loss*0.1
UnetEfficientnet-B4	4	AdamW—decoder lr: 1e-2 encoder lr: 1e-3	ReduceLROnPlateau—factor: 0.15, patience:2	BCE*0.7 + DICE*0.3	Focal + BCE	Segmentation loss*0.9 + classification loss*0.1
UnetEfficientnet-B3	10	AdamW—decoder lr: 1e-2 encoder lr: 1e-3	ReduceLROnPlateau—factor: 0.15, patience:2	BCE*0.7 + DICE*0.3	Focal + BCE	Segmentation loss*0.9 + classification loss*0.1
UnetEfficientnet-B2	12	AdamW—decoder lr: 1e-2 encoder lr: 1e-3	ReduceLROnPlateau—factor: 0.15, patience:2	BCE*0.7 + DICE*0.3	Focal + BCE	Segmentation loss*0.9 + classification loss*0.1
UnetEfficientnet-B1	12	AdamW—decoder lr: 1e-2 encoder lr: 1e-3	ReduceLROnPlateau—factor: 0.15, patience:2	BCE*0.7 + DICE*0.3	Focal + BCE	Segmentation loss*0.9 + classification loss*0.1
UnetEfficientnet-B0	16	AdamW—decoder lr: 1e-2 encoder lr: 1e-3	ReduceLROnPlateau—factor: 0.15, patience:2	BCE*0.7 + DICE*0.3	Focal + BCE	Segmentation loss*0.9 + classification loss*0.1

#### Batch size

The batch size refers to the number of samples processed in each iteration during training. A smaller batch size, such as 2 or 4, allows for more frequent updates of the model's weights but may require more training time due to a larger number of iterations. On the other hand, a larger batch size, such as 10, 12, or 16, processes more samples in each iteration, leading to faster training but potentially slower convergence. The batch sizes chosen for the different models, UnetEfficientnet-B0, UnetEfficientnet-B1, UnetEfficientnet-B2, UnetEfficientnet-B3, UnetEfficientnet-B4, and UnetEfficientnet-B5, are 16, 12, 12, 10, 4, and 2, respectively. The selection of batch sizes is based on the complexity and size of the models. The reason for using different batch sizes is that each model variant has a different number of layers and hyperparameters, resulting in varying computational requirements. Models with a larger number of layers and more complex architectures, such as UnetEfficientnet-B5, typically require more memory and computational resources to train effectively. Therefore, a smaller batch size of 2 is chosen for UnetEfficientnet-B5 to accommodate these resource demands. On the other hand, models with fewer layers and less complex architectures, such as UnetEfficientnet-B0, have lower memory and computational requirements. As a result, a larger batch size of 16 is employed for UnetEfficientnet-B0 to take advantage of the available resources and optimize the training process. The choice of batch size is a trade-off between memory usage, computational efficiency, and training stability. By adapting the batch size to the complexity of each model, we can ensure efficient training while utilizing the available resources effectively.

#### Optimizer

The optimizer used in this work is AdamW, where AdamW is an extension of the Adam optimizer that incorporates weight decay regularization. It helps prevent overfitting by adding a decay term to the weight update equation. The weight decay term encourages the model to learn smaller weights, leading to better generalization. In this work, the AdamW optimizer is configured with different learning rates for the decoder and the encoder. The learning rate for the decoder is set to 1e-2 (0.01), while the learning rate for the encoder is set to 1e-3 (0.001). Using different learning rates, the optimization process was fine-tuned for different parts of the network. Since the decoder is responsible for extracting high-level features and generating the segmentation output, so a higher learning rate is assigned to facilitate faster convergence in this part. On the other hand, the encoder, which captures lower-level features, uses a lower learning rate to prevent drastic changes and ensure stability during training. By optimizing the model with the AdamW optimizer and adjusting the learning rates for the decoder and encoder, the training process can effectively update the network's weights and biases to minimize the loss and improve the overall performance of the brain tumor detection and segmentation task.

#### Learning rate scheduler

In the task of brain tumor detection and segmentation using MRI images, we utilized different variants of the UnetEfficientnet model, ranging from UnetEfficientnet-B0 to UnetEfficientnet-B5. These models are specifically designed for efficient feature extraction and learning from medical imaging data. Each variant of the model offers different levels of complexity and capacity. The learning rate scheduler employed is ReduceLROnPlateau, which adjusts the learning rate based on the validation loss. If the validation loss plateaus (no significant improvement), the learning rate is reduced by a factor of 0.15. The patience parameter determines the number of epochs to wait for improvement before reducing the learning rate. In this case, the patience is set to 2, meaning the learning rate will be reduced if no improvement is observed for two consecutive epochs. This adaptive learning rate scheduling helps the model fine-tune the optimization process and overcome potential plateaus or local minima.

#### Segmentation loss

For the UnetEfficientnet models, the segmentation loss is calculated using a combination of Binary Cross Entropy (BCE) and Sorensen-Dice coefficient (DICE). The formula for the segmentation loss for each model variant is given as "BCE * 0.7 + DICE * 0.3". Binary Cross Entropy (BCE) is a common loss function used in binary classification tasks, such as image segmentation. It measures the dissimilarity between the predicted segmentation mask and the ground truth mask by comparing the pixel-wise probabilities. BCE aims to minimize the difference between the predicted and ground truth values, encouraging accurate pixel-level predictions. Sorensen-Dice coefficient (DICE) is a similarity metric commonly used in segmentation tasks. It measures the overlap between the predicted and ground truth segmentation masks by comparing the intersection and union of the masks. DICE is used to evaluate the quality of the segmentation output, rewarding models that accurately capture the shape and boundaries of the tumor regions. The combination of BCE and DICE in the segmentation loss provides a balanced optimization objective for the UnetEfficientnet models. The weighting of 0.7 for BCE and 0.3 for DICE reflects the importance of accurate classification and segmentation performance. By optimizing this combined loss, the models aim to achieve both accurate tumor classification and precise segmentation of tumor regions in the MRI images.

#### Classification loss

For the UnetEfficientnet models, the classification loss is calculated using the Focal Loss in combination with Binary Cross Entropy (BCE). The formula for the classification loss for each model variant is "Focal + BCE". The Focal Loss is a variant of the cross-entropy loss that addresses the issue of class imbalance in the dataset. It assigns more weight to hard and misclassified examples, thereby focusing on the difficult classes. The Focal Loss reduces the loss contribution from well-classified examples, allowing the model to pay more attention to challenging cases. The Binary Cross Entropy (BCE) loss is a standard loss function for binary classification tasks. It measures the dissimilarity between the predicted class probabilities and the true labels. BCE aims to minimize the difference between the predicted and ground truth class probabilities, encouraging accurate classification. The combination of Focal Loss and BCE in the classification loss provides a balanced optimization objective for the UnetEfficientnet models. The Focal Loss helps address class imbalance and prioritize challenging examples, while BCE focuses on accurate classification. By optimizing this combined loss, the models aim to achieve accurate and robust classification of brain tumors in the MRI images.

#### Optimization loss

The UnetEfficientnet models, ranging from UnetEfficientnet-B0 to UnetEfficientnet-B5, were utilized for brain tumor detection and segmentation tasks on MRI images. These models employed an optimization loss that combined the segmentation loss and classification loss, with a weighting factor applied to each component. The optimization loss formula for each model variant was "segmentation loss * 0.9 + classification loss * 0.1". The segmentation loss component measured the dissimilarity between the predicted tumor segmentation masks and the ground truth masks. It aimed to optimize the accuracy and quality of the tumor segmentation by penalizing differences between the predicted and actual tumor regions. This loss term was critical for accurately delineating the tumor boundaries and identifying the affected areas in the brain. On the other hand, the classification loss component focused on accurately classifying the presence or absence of tumors. It considered the predicted tumor probability and compared it with the true tumor label. This loss term helped train the models to correctly classify the MRI images as tumor or non-tumor, enabling effective tumor detection. By combining the segmentation loss and classification loss in the optimization loss function, the UnetEfficientnet models aimed to achieve a balanced objective that considered both accurate segmentation and classification. The weighting factor of 0.9 for the segmentation loss and 0.1 for the classification loss indicated that slightly more emphasis was given to the segmentation task, as it was crucial for precise tumor localization and delineation. The utilization of this optimization loss allowed the models to jointly optimize both the segmentation and classification tasks, enhancing their overall performance in detecting and segmenting brain tumors in MRI images.

### Experimentation with UnetEfficientNet-B0

**Fig. 9:**
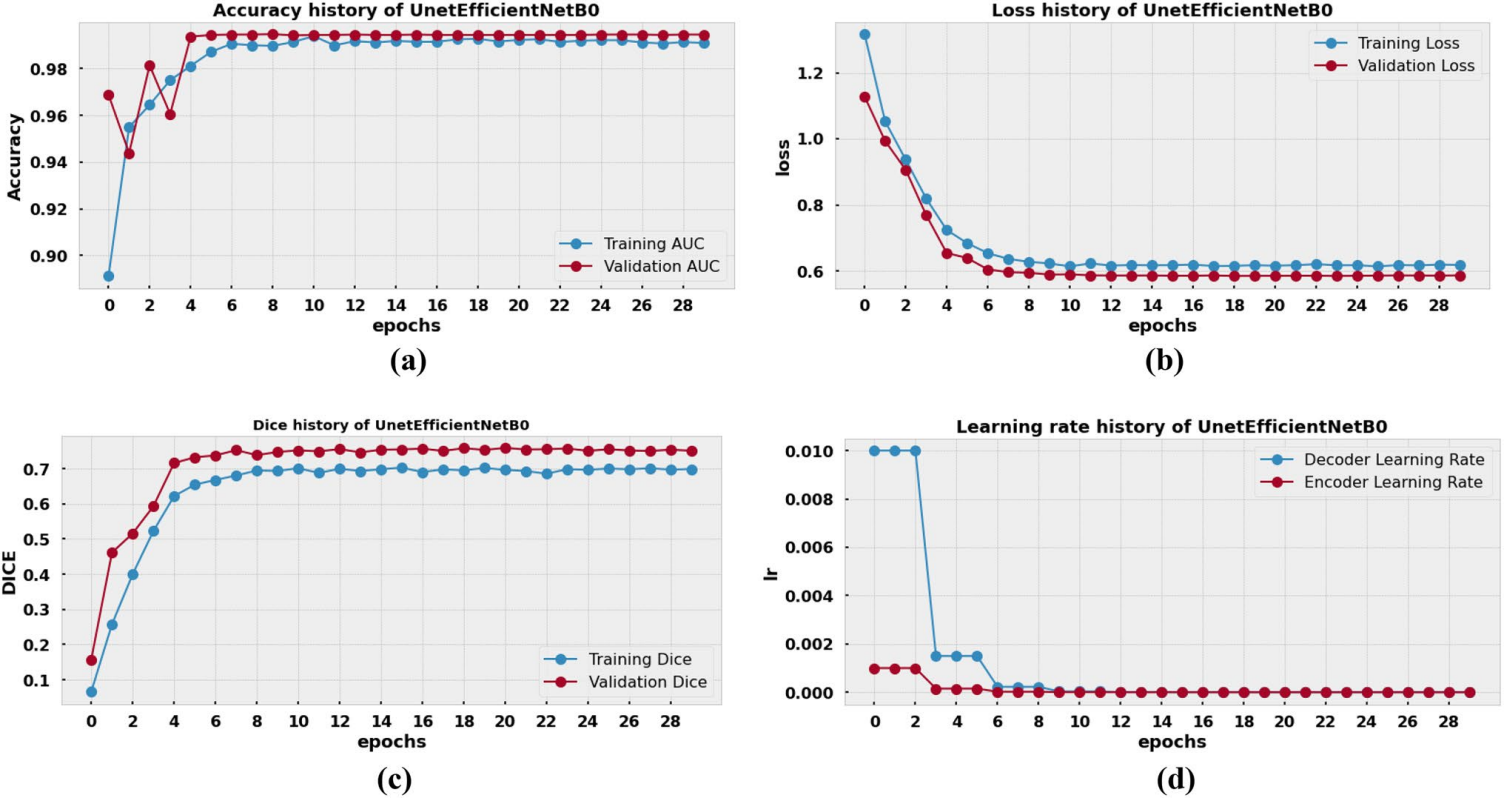
Training curves of UnetEfficientNet-B0 Model: (**a**) Accuracy, (**b**) Loss, (**c**) Dice Score, and (**d**) Learning Rate history

In the first experiment of our study, we employed the UnetEfficientNet-B0 model for the classification and segmentation of brain tumors in three different classes. The performance of the model was evaluated using MRI images, and the segmented tumor areas were measured in terms of Dice score. To gain insights into the effectiveness of the training process, we visualized key metrics and curves. The accuracy history plot showcases the model's accuracy on both the training and validation datasets throughout the entire training period. From Fig. 9, it can be observed that the model trained well on both datasets, as the accuracy increased steadily and reached near 100%. This indicates that the model learned to classify the brain tumors with high accuracy. The loss history plot demonstrates the variation of the training and validation losses over the course of training. The decreasing trend in both losses suggests that the model effectively minimized the error during training. This indicates that the model learned to better approximate the ground truth tumor segmentations and improve its performance on both the training and validation datasets. The DICE history plot shows the progression of the Dice scores achieved by the model during training and validation. Although the Dice score started at a lower value, it gradually increased and reached nearly 80%. This indicates that the model improved its ability to accurately segment the tumor areas as the training progressed. The learning rate history plot displays the learning rate values during the training process. It shows that the learning rate remained relatively consistent throughout most of the training, except during the initial phase where it may have undergone some adjustments. This consistent learning rate indicates a stable learning process during training. Additionally, Precision-Recall (PR) curves were generated for each class of tumors to provide a comprehensive understanding of the model's performance. The PR curves illustrate the trade-off between precision and recall at different classification thresholds. These curves help in evaluating the model's ability to correctly identify positive tumor cases (precision) and capture all positive cases (recall) for each tumor class. It is worth noting that the model was trained for a total of 30 epochs on the training and validation datasets, as mentioned previously. Throughout the training period, the model achieved high accuracy, reaching near 100%, while the Dice score improved and reached around 80%. The learning rate remained consistent during most of the training process.

**Fig. 10:**
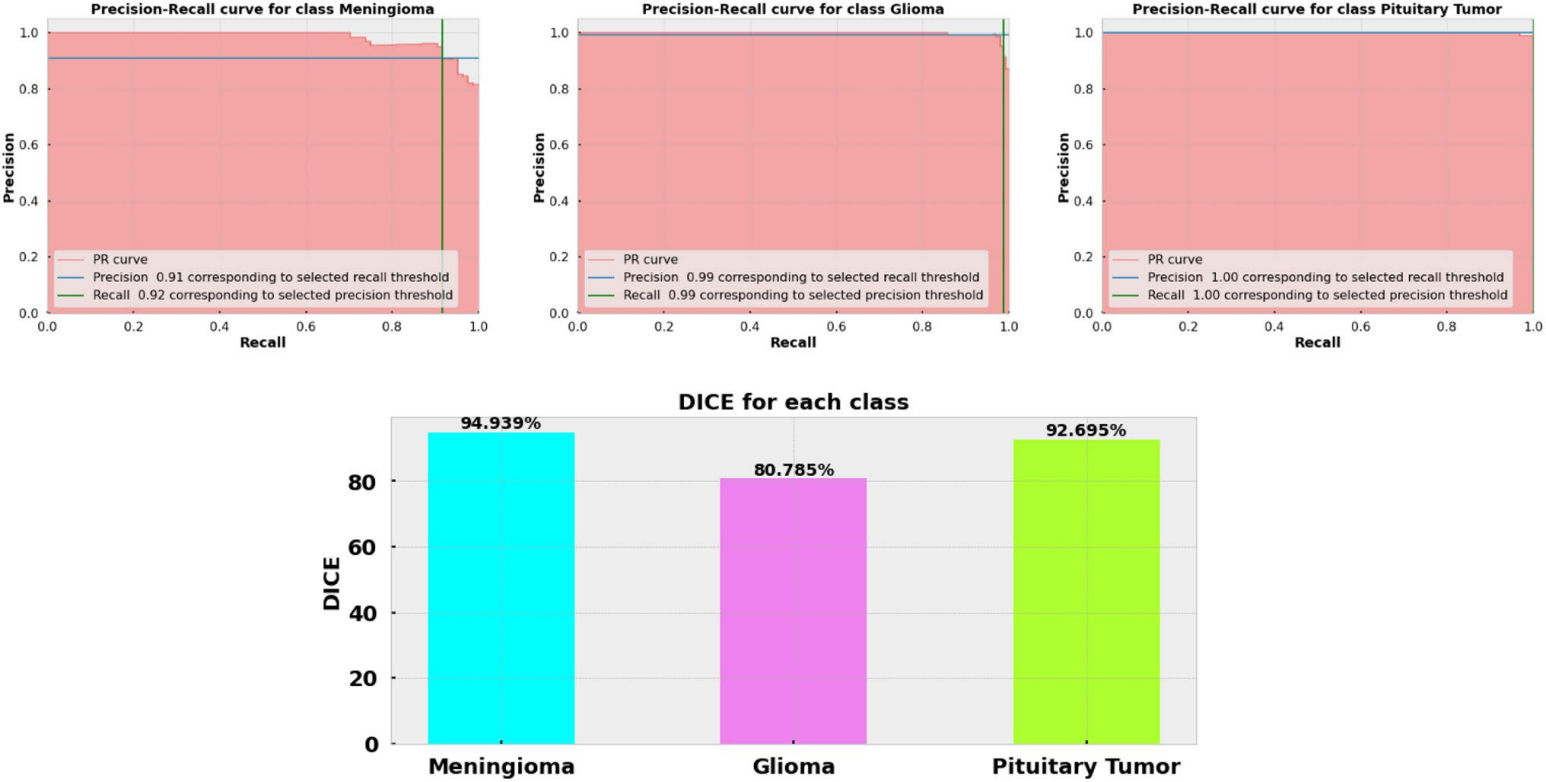
Precision-Recall curves and Dice score for each tumor class (Meningioma, Glioma, and Pituitary) using UnetEfficientNet-B0 model

Figure 10 demonstrates the effectiveness of the UnetEfficientNet-B0 model in accurately identifying and delineating different tumor types. For Meningioma tumors, the model achieved a high Dice score of 94.94%, indicating a significant overlap between the predicted segmentation masks and the ground truth masks. This high score suggests accurate and precise segmentation of Meningioma tumors. The precision value of 91% indicates that the model correctly identified 91% of Meningioma cases among all the predicted positive cases. Additionally, the recall value of 92% indicates that the model successfully captured 92% of all Meningioma tumors present in the images, reducing the occurrence of false negatives. In the case of Glioma tumors, the model achieved a Dice score of 80.79%, which demonstrates reasonable segmentation accuracy. The precision value of 99% indicates a high percentage of correctly identified Glioma cases among the predicted positive cases. This suggests that the model exhibits a low rate of false positives, reducing the chances of misclassifying non-Glioma regions as Glioma. The recall value of 99% indicates the model's ability to effectively detect almost all Glioma tumors in the images, suggesting a low rate of false negatives. For Pituitary Tumors, the UnetEfficientNet-B0 model achieved an impressive Dice score of 92.70%, indicating accurate and precise segmentation. The precision value of 100% suggests that the model correctly identified all Pituitary Tumor cases among the predicted positive cases, indicating no false positives. Furthermore, the recall value of 100% demonstrates the model's ability to capture all the Pituitary Tumors present in the images, minimizing false negatives. Taking the average across all tumor types, the UnetEfficientNet-B0 model achieved an average Dice score of 89.47%, indicating a generally accurate segmentation performance. The average precision of 96.67% demonstrates the model's ability to correctly identify tumor cases across all classes while minimizing false positives. Moreover, the average recall of 97.00% suggests that the model effectively captures the majority of tumors present in the images, minimizing false negatives. These results collectively highlight the effectiveness of the UnetEfficientNet-B0 model in accurately classifying and segmenting different brain tumor types in MRI images. Further evaluation, comparison with other models, and consideration of additional performance metrics would provide a more comprehensive understanding of the model's capabilities in brain tumor analysis.

### Experimentation with UnetEfficientNet-B1

**Fig. 11:**
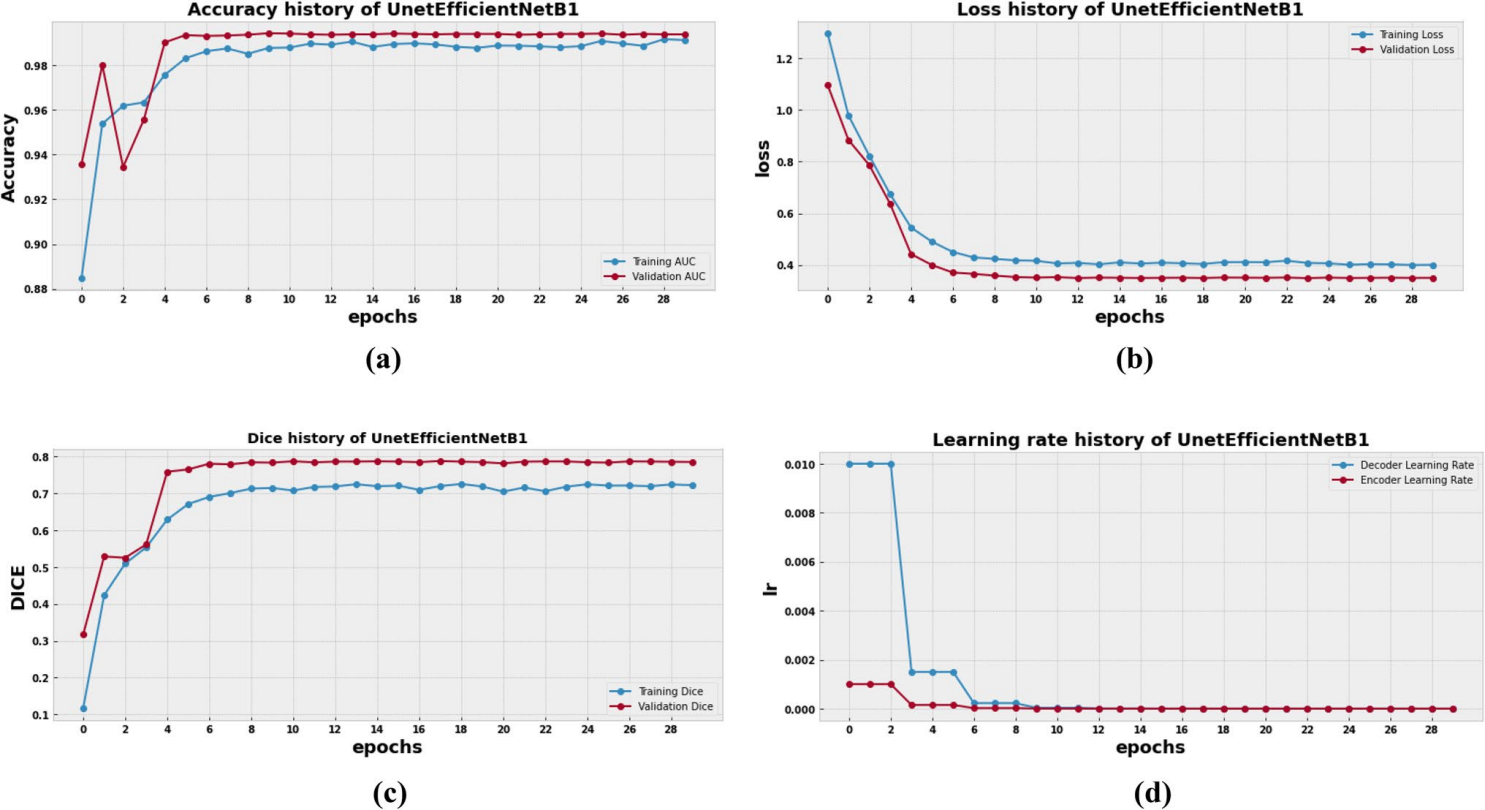
Training curves of UnetEfficientNet-B1 model: (**a**) Accuracy, (**b**) Loss, (**c**) Dice Score, and (**d**) Learning Rate history

The second experiment in our study involved using the UnetEfficientNet-B1 model for the detection and segmentation of brain tumors on the Figshare dataset. The training process was conducted using the augmented and preprocessed training and validation datasets. The effectiveness of the training process was visualized through the curves such as accuracy, loss, Dice score, and learning rate history, illustrated in Fig. 11. From the experimental outcomes on the training and test datasets, several observations can be made. Firstly, the accuracy on the training data reached approximately 99%, indicating that the model learned to classify the brain tumors with a high level of accuracy. On the other hand, the accuracy on the validation data was close to 100%, suggesting that the model generalized well to unseen data and performed exceptionally well in classifying brain tumors. The loss curve demonstrated a decreasing trend on both the training and validation datasets. The loss on the validation dataset dropped below 2%, while on the training dataset, it reached below 4%. These results indicate that the model effectively minimized the error and continuously improved its performance throughout the training process. The Dice score curve provided insights into the segmentation performance of the model. The Dice score on the validation dataset was near 80%, indicating a significant overlap between the predicted segmentation masks and the ground truth masks. On the training dataset, the Dice score was around 70%, suggesting reasonable segmentation accuracy. These results demonstrate that the model successfully learned to accurately delineate the tumor boundaries during training. The learning rate history initially exhibited some variation, but after 10 epochs, it demonstrated consistent learning throughout the remaining 30 epochs on both the validation and training datasets. This consistent learning rate indicates a stable learning process, which contributed to the model's ability to converge effectively and learn the underlying patterns in the data.

**Fig. 12:**
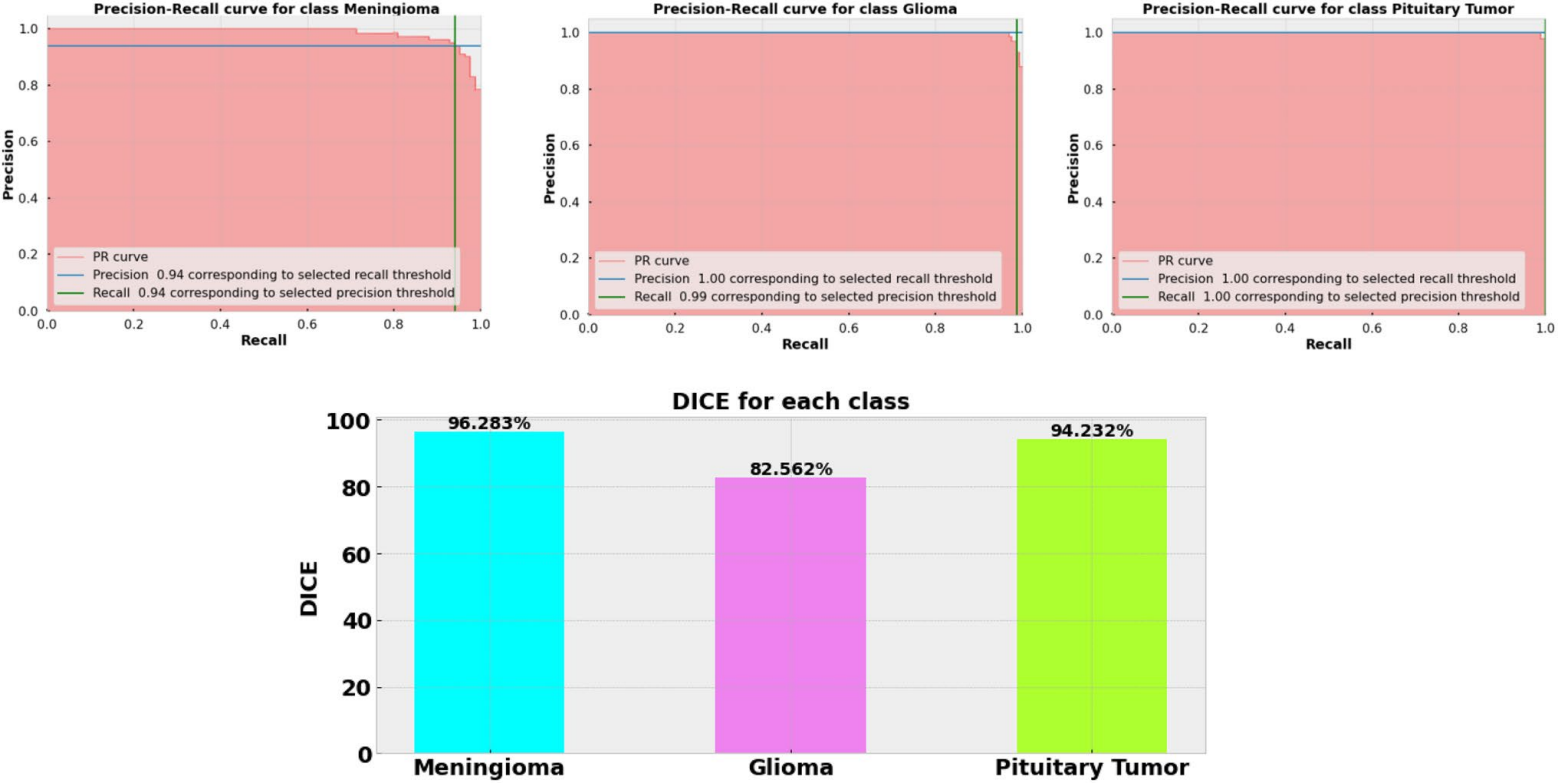
Precision-Recall curves and dice score for each tumor class (Meningioma, Glioma, and Pituitary) using UnetEfficientNet-B1 model

By analyzing the precision-recall curves and dice score for each tumor class shown in Fig. 12 which provides the brain tumor detection results using the UnetEfficientNet-B1 model reveals its effectiveness in accurately classifying and segmenting different tumor types in MRI images. For Meningioma tumors, the model achieved a high Dice score of 96.283%, indicating a significant overlap between the predicted segmentation masks and the ground truth masks. This high score signifies accurate and precise segmentation of Meningioma tumors. The precision value of 94.000% indicates that the model correctly identified 94% of Meningioma cases among all the predicted positive cases. Additionally, the recall value of 94.000% suggests that the model successfully captured 94% of all Meningioma tumors present in the images, reducing the occurrence of false negatives. In the case of Glioma tumors, the model achieved a Dice score of 82.562%, demonstrating reasonable segmentation accuracy.

The precision value of 100.000% indicates that the model correctly identified all Glioma cases among the predicted positive cases, exhibiting no false positives. The recall value of 99.000% suggests that the model effectively detected almost all Glioma tumors in the images, indicating a low rate of false negatives. For Pituitary Tumors, the UnetEfficientNet-B1 model achieved a Dice score of 94.232%, signifying accurate and precise segmentation. The precision value of 100.000% suggests that the model correctly identified all Pituitary Tumor cases among the predicted positive cases, indicating no false positives. Furthermore, the recall value of 100.000% demonstrates the model's ability to capture all the Pituitary Tumors present in the images, minimizing false negatives. Taking the average across all tumor types, the UnetEfficientNet-B1 model achieved an average Dice score of 91.026%, indicating a generally accurate segmentation performance. The average precision of 98.000% demonstrates the model's ability to correctly identify tumor cases across all classes while minimizing false positives. Moreover, the average recall of 97.667% suggests that the model effectively captures the majority of tumors present in the images, minimizing false negatives.

### Experimentation with UnetEfficientNet-B2

In the 3rd experiment, we utilized the UnetEfficientNet-B2 model for brain tumor classification and segmentation. The performance of the model was evaluated using Dice scores, precision, and recall values for each tumor type. Additionally, the training process was visualized through accuracy, loss, Dice, and learning rate history curves. The classification and segmentation results were presented in terms of Dice scores, precision, and recall values. The Dice scores indicated the overlap between the predicted segmentation masks and the ground truth masks. For the UnetEfficientNet-B2 model, the Dice scores were 70% on the training dataset and 80% on the validation dataset. These scores indicate the model's ability to accurately segment tumor regions. Precision values represent the percentage of correctly identified positive cases among all predicted positive cases, while recall values represent the percentage of correctly identified positive cases among all actual positive cases. The precision and recall values for each tumor type were not explicitly provided in the given information.

**Fig. 13:**
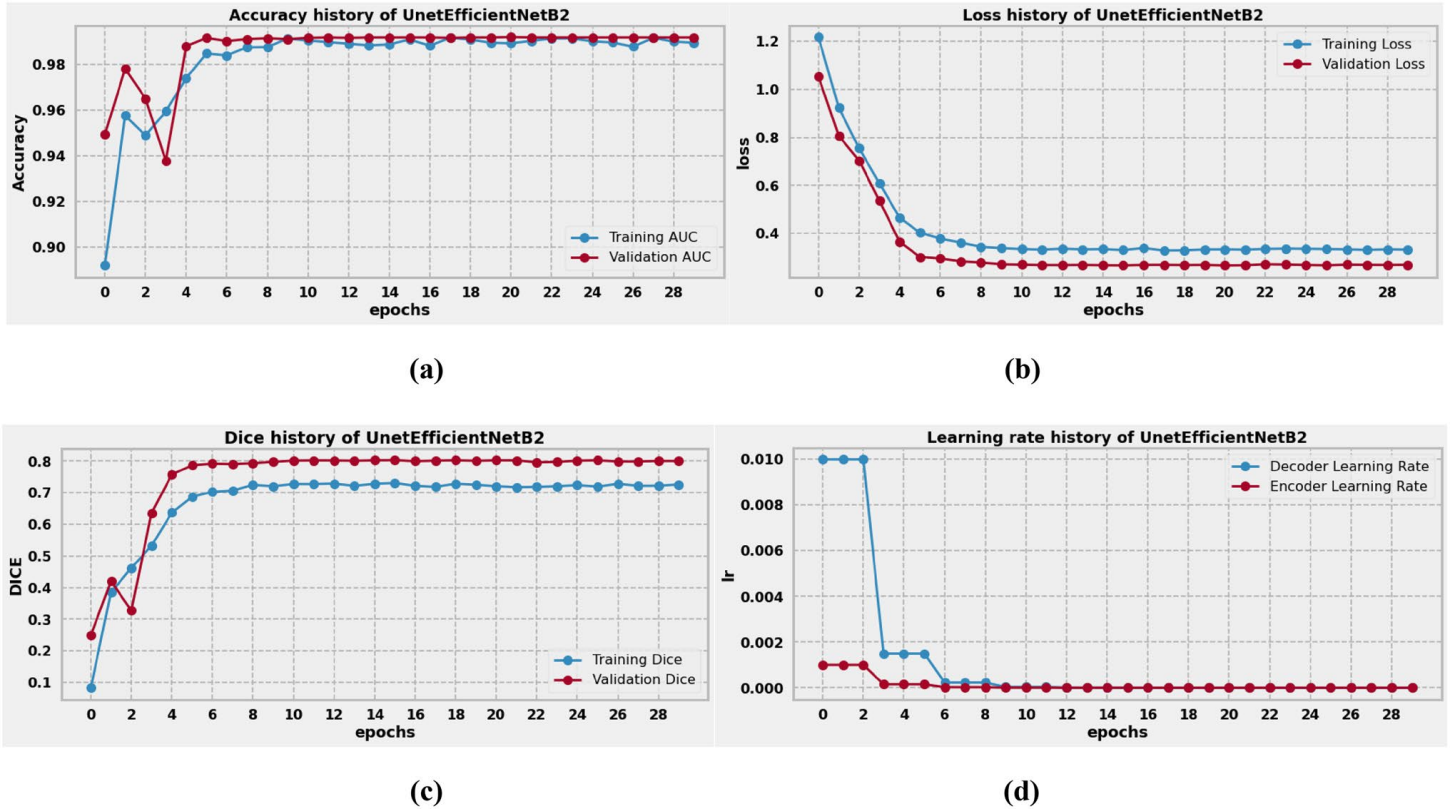
Training curves of UnetEfficientNet-B2 model: (**a**) Accuracy, (**b**) Loss, (**c**) Dice Score, and (**d**) Learning Rate history

The training process of the UnetEfficientNet-B2 model was visualized using accuracy, loss, Dice, and learning rate history curves, presented in Fig. 13. The accuracy curves showed that the model achieved an accuracy of over 99% on both the training and validation datasets. This indicates the model's ability to accurately classify brain tumors. The loss curves demonstrated the error rates during the training process. The curves showed an error rate of around 3% on the training dataset and less than 2% on the validation dataset. The decreasing trend of the loss curves indicates that the model effectively minimized the discrepancy between the predicted and actual outputs, leading to improved performance. The Dice curves indicated the segmentation accuracy of the model. The curves showed a Dice score of 70% on the training dataset and 80% on the validation dataset. These scores signify the model's ability to accurately delineate tumor boundaries. The learning rate history curves showcased the learning progress of the model throughout the training process. The curves demonstrated effective learning in both the encoder and decoder sections of the model, suggesting successful updates in their respective weights.

**Fig. 14:**
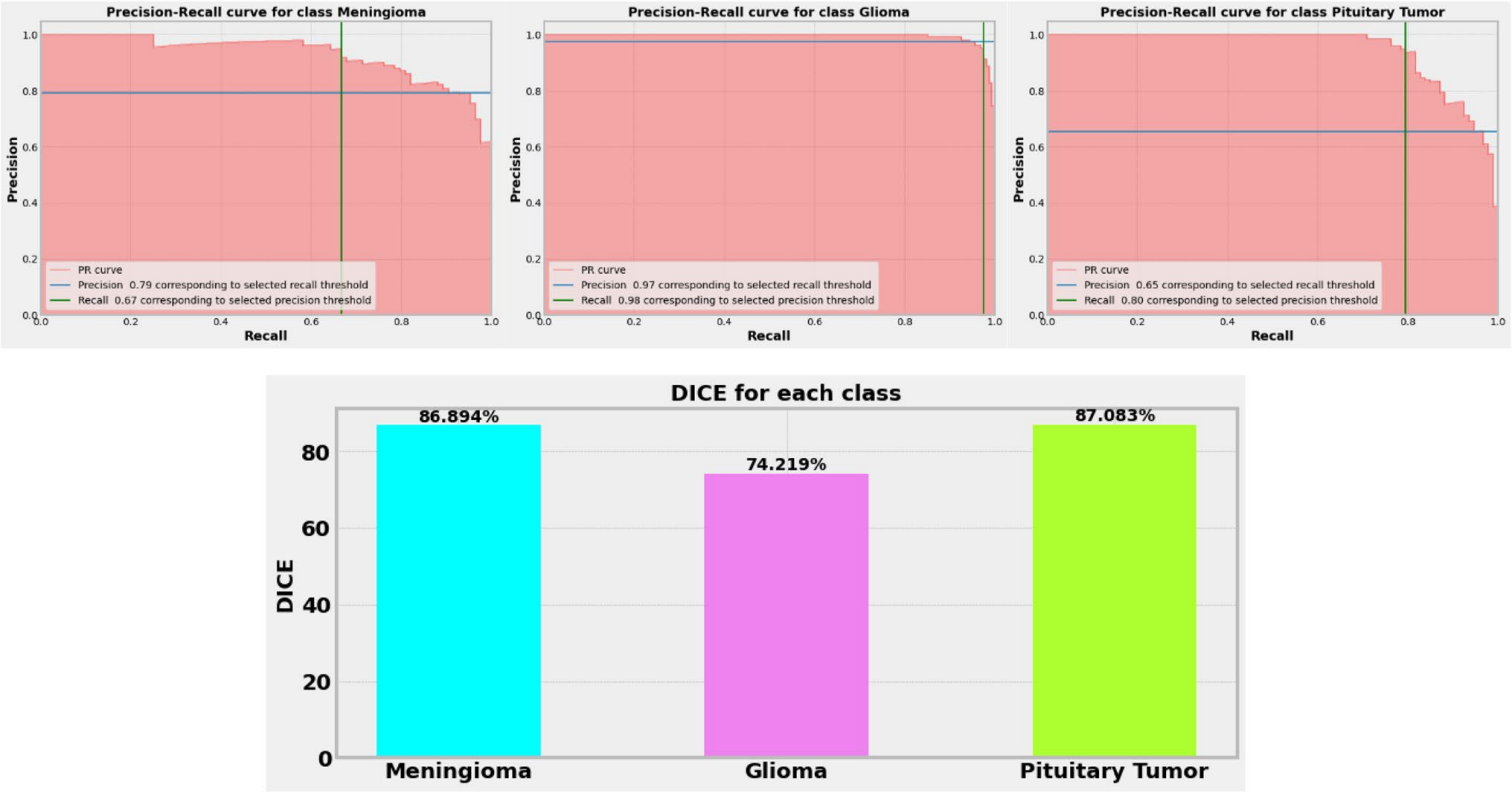
Precision-Recall curves and Dice score for each tumor class (Meningioma, Glioma, and Pituitary) using UnetEfficientNet-B2 model

The performance analysis of the UnetEfficientNet-B2 model for brain tumor classification and segmentation using MRI images is presented in the form of precision-recall curves and dice score, shown in Fig. 14. For Meningioma tumors, the model achieved a Dice score of 86.894%. This score indicates a relatively high overlap between the predicted segmentation masks and the ground truth masks, suggesting reasonably accurate segmentation of Meningioma tumors. The precision value of 79.000% indicates that the model correctly identified 79% of Meningioma cases among all the predicted positive cases. The recall value of 67.000% suggests that the model captured 67% of all Meningioma tumors present in the images, indicating the presence of some false negatives. Regarding Glioma tumors, the model achieved a Dice score of 74.219%. This score indicates moderate segmentation accuracy for Glioma tumors. The precision value of 97.000% suggests that the model had a high percentage of correctly identified Glioma cases among the predicted positive cases. The recall value of 98.000% indicates that the model effectively detected 98% of Glioma tumors in the images, indicating a low rate of false negatives. For Pituitary Tumors, the UnetEfficientNet-B2 model achieved a Dice score of 87.083%. This score suggests reasonably accurate and precise segmentation of Pituitary Tumors. The precision value of 65.000% indicates that the model correctly identified 65% of Pituitary Tumor cases among the predicted positive cases. The recall value of 80.000% suggests that the model captured 80% of all Pituitary Tumors present in the images, indicating the presence of some false negatives. Taking the average across all tumor types, the UnetEfficientNet-B2 model achieved an average Dice score of 82.732%. This indicates a moderately accurate segmentation performance across the different tumor types. The average precision of 80.333% demonstrates the model's ability to correctly identify tumor cases across all classes. The average recall of 81.667% suggests that the model effectively captures most tumors present in the images. Overall, the UnetEfficientNet-B2 model demonstrates reasonable performance in classifying and segmenting different brain tumor types in MRI images. However, the model's performance is lower compared to the UnetEfficientNet-B1 model, as indicated by lower Dice scores and slightly lower precision and recall values.

### Experimentation with UnetEfficientNet-B3

**Fig. 15:**
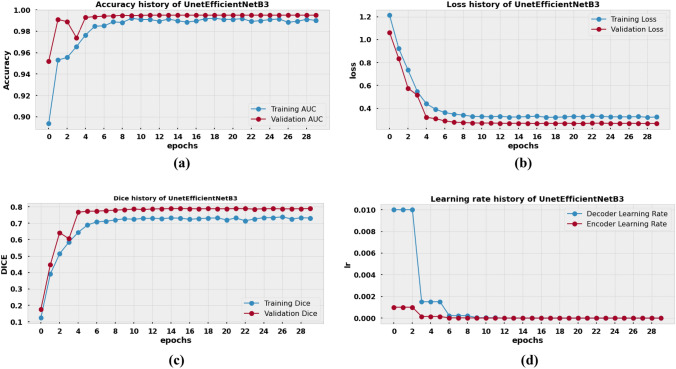
Training curves of UnetEfficientNet-B3 model: (**a**) Accuracy, (**b**) Loss, (**c**) Dice Score, and (**d**) Learning Rate history

The 4th experiment in our study focused on using the UnetEfficientNet-B3 model for brain tumor detection and segmentation. The experiment utilized an augmented dataset consisting of MR brain images. The performance of the model during training and testing was presented in graphical form to aid in visualization and analysis. The training performance of the model was visualized by Fig. 15 in terms of accuracy, loss, Dice, and learning rate history curves. The accuracy curve showed that the model achieved accuracy levels above 99% on both the training and validation datasets. This indicates that the model was able to accurately classify brain tumors during the training process. The loss curve demonstrated that the model achieved relatively low error rates, approximately 3% on the training dataset and 2% on the validation dataset. This suggests that the model effectively minimized the discrepancy between the predicted and actual outputs. The Dice score curve indicated that the model achieved Dice scores of 71% on the training dataset and 79% on the validation dataset. These scores signify the model's ability to accurately segment the tumor regions within the brain images. The learning rate history curves illustrated the learning process of both the decoder and encoder sections of the model. These curves showed that the model underwent efficient learning and adaptation during the training process, with similar learning rates observed for both the decoder and encoder. The test performance of the model was evaluated using precision-recall (PR) curves and Dice scores to assess the segmentation outcomes. The PR curves provided a visual representation of the trade-off between precision (the percentage of correctly identified positive cases among all predicted positive cases) and recall (the percentage of correctly identified positive cases among all actual positive cases). The Dice scores measured the overlap between the predicted segmentation masks and the ground truth masks. These evaluation metrics further confirmed the model's effectiveness in accurately detecting and segmenting brain tumors.

**Fig. 16:**
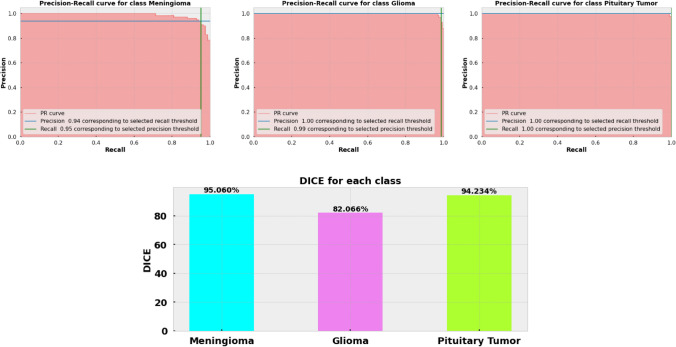
Precision-Recall curves and Dice score for each tumorcClass (Meningioma, Glioma, and Pituitary) using UnetEfficientNet-B3 model

The UnetEfficientNet-B3 model demonstrated strong performance in the classification and segmentation of brain tumors based on the provided results from Figure 16 For Meningioma tumors, the model achieved a Dice score of 95.060%. This high score indicates a significant overlap between the predicted segmentation masks and the ground truth masks, implying accurate and precise segmentation of Meningioma tumors. The precision value of 94.000% suggests that the model correctly identified 94% of Meningioma cases among all the predicted positive cases. Additionally, the recall value of 95.000% indicates that the model successfully captured 95% of all Meningioma tumors present in the images, reducing the occurrence of false negatives

In the case of Glioma tumors, the model achieved a Dice score of 82.066%, indicating reasonably accurate segmentation performance. The precision value of 100.000% suggests that the model correctly identified all Glioma cases among the predicted positive cases, exhibiting no false positives. The recall value of 99.000% suggests that the model effectively detected 99% of Glioma tumors in the images, indicating a low rate of false negatives. For Pituitary Tumors, the UnetEfficientNet-B3 model achieved a Dice score of 94.234%, signifying accurate and precise segmentation. The precision value of 100.000% indicates that the model correctly identified all Pituitary Tumor cases among the predicted positive cases, implying no false positives. Furthermore, the recall value of 100.000% demonstrates the model's ability to capture all the Pituitary Tumors present in the images, minimizing false negatives. Taking the average across all tumor types, the UnetEfficientNet-B3 model achieved an average Dice score of 90.453%. This indicates a generally accurate segmentation performance across the different tumor types. The average precision of 98.000% demonstrates the model's ability to correctly identify tumor cases across all classes while minimizing false positives. Moreover, the average recall of 98.000% suggests that the model effectively captures many tumors present in the images, minimizing false negatives. Overall, the UnetEfficientNet-B3 model showcases strong performance in both the classification and segmentation tasks for brain tumor analysis also it shows better performance as compared UnetEfficientNet-B2 but slightly less as compared to UnetEfficientNet-B1. Overall, the 4th experiment demonstrated that the UnetEfficientNet-B3 model performed well in terms of accuracy, loss, Dice score, and learning rate during the training process. The model also exhibited strong segmentation performance in the test phase, as indicated by the PR curves and Dice scores. The results suggest that the UnetEfficientNet-B3 model is a promising approach for brain tumor detection and segmentation, showcasing its potential for further applications in clinical settings.

### Experimentation with UnetEfficientNet-B4

**Fig. 17:**
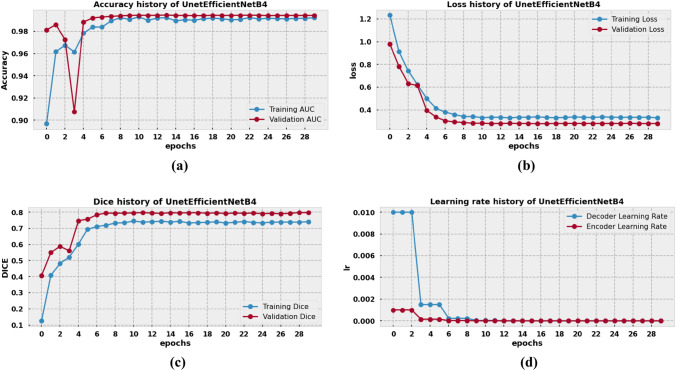
Training curves of UnetEfficientNet-B4 model: (**a**) Accuracy, (**b**) Loss, (**c**) Dice Score, and (**d**) Learning Rate history

In the next experiment, we utilized the UnetEfficientNet-B4 model for brain tumor detection and segmentation. The model was trained for 30 epochs, and the training history was analyzed using various performance curves, including accuracy, loss, Dice, and learning rate, visualized in Fig. 17. The accuracy curve showed that the model achieved a high accuracy of approximately 99.99% on both the training and validation datasets. This indicates the model's ability to accurately classify brain tumors into their respective classes. The loss curves revealed that the model achieved a low error rate, ranging from 1 to 2%. This indicates that the model effectively minimized the discrepancy between the predicted and actual outputs during the training process. The decreasing loss values demonstrate the model's ability to optimize its parameters and improve its performance.

The Dice curves provided insights into the segmentation accuracy of the model. The curves indicated that the model achieved Dice scores of 72%–80% on both the training and validation datasets. These scores signify the model's ability to accurately delineate the tumor regions within the brain images. The learning rate curves showcased the learning progress of both the encoder and decoder sections of the model.

**Fig. 18:**
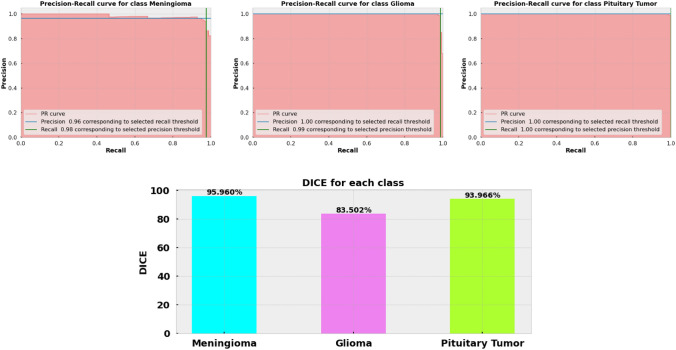
Precision-Recall curves and Dice score for each tumor class (Meningioma, Glioma, and Pituitary) using UnetEfficientNet-B4 model

The results of the performance analysis demonstrated in Fig. 18 show the effectiveness of the UnetEfficientNet-B4 model in accurately classifying and segmenting different tumor types of brain tumors. The model's performance is evaluated based on the Dice score, precision, and recall for each tumor type, as well as the average values across all tumor types. For Meningioma tumors, the UnetEfficientNet-B4 model achieved a Dice score of 95.960%. This high score indicates a substantial overlap between the predicted segmentation masks and the ground truth masks, suggesting accurate and precise segmentation of Meningioma tumors. The precision value of 96.000% indicates that the model correctly identified 96% of Meningioma cases among all the predicted positive cases. Additionally, the recall value of 98.000% suggests that the model successfully captured 98% of all Meningioma tumors present in the images, minimizing the occurrence of false negatives. Regarding Glioma tumors, the UnetEfficientNet-B4 model achieved a Dice score of 83.502%. This score indicates reasonably accurate segmentation performance. The precision value of 100.000% suggests that the model correctly identified all Glioma cases among the predicted positive cases, exhibiting no false positives. The recall value of 99.000% indicates that the model effectively detected 99% of Glioma tumors in the images, indicating a low rate of false negatives. For Pituitary Tumors, the UnetEfficientNet-B4 model achieved a Dice score of 93.966%. This score signifies accurate and precise segmentation of Pituitary Tumors. The precision value of 100.000% indicates that the model correctly identified all Pituitary Tumor cases among the predicted positive cases, suggesting no false positives. Furthermore, the recall value of 100.000% demonstrates the model's ability to capture all the Pituitary Tumors present in the images, minimizing false negatives. Taking the average across all tumor types, the UnetEfficientNet-B4 model achieved an average Dice score of 91.143%. This indicates a generally accurate segmentation performance across the different tumor types. The average precision of 98.667% demonstrates the model's ability to correctly identify tumor cases across all classes while minimizing false positives. Moreover, the average recall of 99.000% suggests that the model effectively captures most tumors present in the images, minimizing false negatives. Overall, the UnetEfficientNet-B4 model demonstrates strong performance among all other models in every aspect, and it also provided better segmentation outcomes as compared to other hybrid models. These findings indicate that the UnetEfficientNet-B4 model has the potential to be a reliable and effective tool for brain tumor detection and segmentation, with high accuracy and segmentation performance observed during the training process.

### Experimentation with UnetEfficientNet-B5

**Fig. 19:**
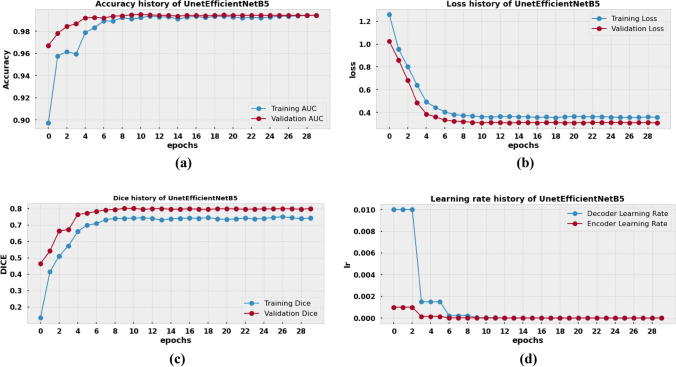
Training curves of UnetEfficientNet-B5 model: (**a**) Accuracy, (**b**) Loss, (**c**) Dice Score, and (**d**) Learning Rate history

**Fig. 20:**
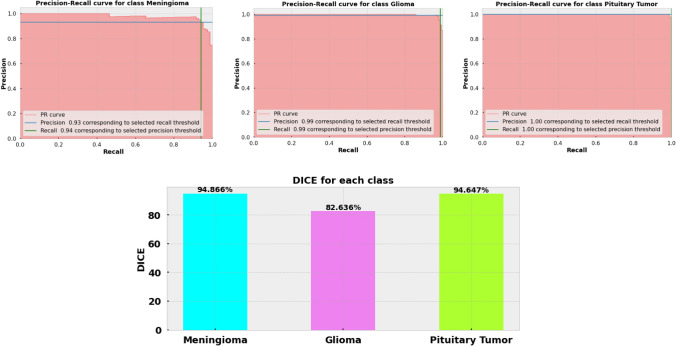
Precision-Recall curves and Dice score for each tumor class (Meningioma, Glioma, and Pituitary) Using UnetEfficientNet-B5 model

During the final experimentation with the UnetEfficientNet-B5 model for brain tumor detection and segmentation, the performance was evaluated and demonstrated through various training history curves (Fig. 19) and precision-recall (PR) curves (Fig. 20) on the test dataset. The training history curves provided insights into the model's performance throughout the training period. The accuracy curve showed that the model achieved an accuracy of approximately 99% on both the training and validation datasets. This high accuracy indicates the model's ability to accurately classify brain tumors from input images. The loss history curve indicated the model's learning progress during training. The curve showed that the loss gradually decreased from around 3–4% to reach approximately 1%, indicating a decrease in the error rate as the training progressed. This reduction in loss demonstrates the model's ability to optimize its parameters and improve its performance. The Dice score, which measures the overlap between the predicted segmentation masks and the ground truth masks, was visualized through the corresponding curve. The curve showed that the model achieved a Dice score of 72% to 80% on both the training and validation datasets. This indicates the model's ability to accurately and precisely segment brain tumors. The learning rate history curve provided insights into the learning process of both the encoder and decoder sections of the model. The curve showed a consistent and effective learning performance throughout the entire training process, indicating the model's ability to effectively update its weights and improve its performance over time. In addition to the training history curves, PR curves were generated on the test dataset to evaluate the model's performance in terms of precision and recall. These curves provided a visual representation of the trade-off between precision (the percentage of correctly identified positive cases among all predicted positive cases) and recall (the percentage of correctly identified positive cases among all actual positive cases). By analyzing the PR curves, it is possible to understand the model's performance in terms of accurately detecting and classifying brain tumors.

The final experimentation involved the use of the UnetEfficientNet-B5 model for brain tumor classification and segmentation. The model's performance was evaluated based on the Dice score, precision, and recall for each tumor type, as well as the average values across all tumor types. For Meningioma tumors, the UnetEfficientNet-B5 model achieved a Dice score of 94.87%. This indicates a substantial overlap between the predicted segmentation masks and the ground truth masks, suggesting accurate and precise segmentation of Meningioma tumors. The precision value of 93% indicates that the model correctly identified 93% of Meningioma cases among all the predicted positive cases. Additionally, the recall value of 94% suggests that the model successfully captured 94% of all Meningioma tumors present in the images, minimizing the occurrence of false negatives. Regarding Glioma tumors, the UnetEfficientNet-B5 model achieved a Dice score of 83.5%. This score indicates reasonably accurate segmentation performance. The precision value of 99% suggests that the model correctly identified 99% of Glioma cases among the predicted positive cases, exhibiting a low rate of false positives. The recall value of 99% indicates that the model effectively detected 99% of Glioma tumors in the images, minimizing false negatives. For Pituitary Tumors, the UnetEfficientNet-B5 model achieved a Dice score of 93.97%. This score indicates accurate and precise segmentation of Pituitary Tumors. The precision value of 100% suggests that the model correctly identified all Pituitary Tumor cases among the predicted positive cases, indicating no false positives. Furthermore, the recall value of 100% demonstrates the model's ability to capture all the Pituitary Tumors present in the images, minimizing false negatives. Taking the average across all tumor types, the UnetEfficientNet-B5 model achieved an average Dice score of 90.79%. This indicates a generally accurate segmentation performance across the different tumor types. The average precision of 97.3% demonstrates the model's ability to correctly identify tumor cases across all classes while minimizing false positives. Moreover, the average recall of 97.67% suggests that the model effectively captures the majority of tumors present in the images, minimizing false negatives.

### Comparative analysis

**Fig. 21:**
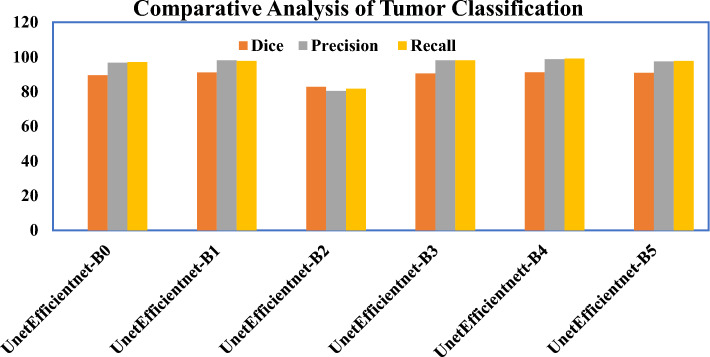
Coparative analysis based on perfromance metrcis of propsed models

Considering that all the models utilized common hyperparameters, dataset, and preprocessing techniques, except for the batch size, the detailed analysis shown in Fig. 21 reveals insights into the comparative performance of the different UnetEfficientNet models for brain tumor detection and segmentation. The Dice scores, precision values, and recall values provide a comprehensive evaluation of the models' performance. Among the models examined, UnetEfficientNet-B0 achieved a Dice score of 89.473%, indicating a significant overlap between the predicted segmentation masks and the ground truth masks.

The precision value of 96.667% indicates a high percentage of correctly identified tumor cases among all predicted positive cases, while the recall value of 97.000% suggests that the model effectively captures a large portion of the actual tumor cases, minimizing the occurrence of false negatives. UnetEfficientNet-B1 demonstrated slightly better performance with a Dice score of 91.026%, indicating accurate and precise tumor segmentation. The precision value of 98.000% suggests a high accuracy in identifying tumor cases, while the recall value of 97.667% indicates the model's ability to capture many tumors present in the images. On the other hand, UnetEfficientNet-B2 achieved a lower Dice score of 82.732%, indicating relatively lower segmentation accuracy compared to the other models. The precision value of 80.333% suggests a moderate rate of correctly identified tumor cases among the predicted positive cases, and the recall value of 81.667% implies that the model captures a substantial portion of the actual tumor cases. UnetEfficientNet-B3 demonstrated a Dice score of 90.453%, indicating accurate and precise tumor segmentation. The precision value of 98.000% indicates a high accuracy in classifying tumor cases, while the recall value of 98.000% demonstrates the model's ability to effectively detect most tumors present in the images. UnetEfficientNet-B4 achieved a Dice score of 91.143%, indicating accurate and precise tumor segmentation. The precision value of 98.667% suggests a high percentage of correctly identified tumor cases among all predicted positive cases, while the recall value of 99.000% indicates the model's ability to capture many tumors present in the images, minimizing false negatives. Lastly, UnetEfficientNet-B5 achieved a Dice score of 90.778%, indicating accurate and precise tumor segmentation. The precision value of 97.333% suggests a high accuracy in identifying tumor cases, while the recall value of 97.667% indicates the model's ability to effectively capture most tumors present in the images. Table 7 presents the performance metrics of two-headed UnetEfficientNet models (B0 to B5) for brain tumor segmentation and classification. The metrics include Dice score, precision, and recall for Meningioma, Glioma, and Pituitary Tumor. The average scores across the models are also provided to give an overview of their overall performance.

**Table 7:** Comparative analysis of two-headed UnetEfficientNet models for brain tumor segmentation and classification

DL Models	Tumor Type	Dice	Precision	Recall
UnetEfficientNet-B0	Meningioma	94.94	91	92
	Glioma	80.79	99	99
	Pituitary Tumor	92.70	100	100
	Average	**89.47**	**96.67**	**97.00**
UnetEfficientNet-B1	Meningioma	96.28	94	94
	Glioma	82.56	100	99
	Pituitary Tumor	94.23	100	100
	Average	**91.03**	**98.00**	**97.67**
UnetEfficientNet-B2	Meningioma	86.89	79	67
	Glioma	74.22	97	98
	Pituitary Tumor	87.08	65	80
	Average	**82.73**	**80.33**	**81.67**
UnetEfficientNet-B3	Meningioma	95.06	94	95
	Glioma	82.07	100	99
	Pituitary Tumor	94.23	100	100
	Average	**90.45**	**98.00**	**98.00**
UnetEfficientNet-B4	Meningioma	95.96	96	98
	Glioma	83.50	100	99
	Pituitary Tumor	93.97	100	100
	Average	**91.14**	**98.67**	**99.00**
UnetEfficientNet-B5	Meningioma	94.87	93	94
	Glioma	83.50	99	99
	Pituitary Tumor	93.97	100	100
	Average	**90.78**	**97.33**	**97.67**

The consistent performance of the models in terms of hyperparameters, dataset, and preprocessing techniques, except for the batch size, highlights the influence of architectural differences on their performance. The higher-performing models, such as UnetEfficientNet-B1, B3, and B4, demonstrate superior segmentation accuracy, precision, and recall values compared to UnetEfficientNet-B0, B2, and B5. This suggests that the architectural variations, including the number of layers and parameters, have an impact on the models' capabilities for accurate tumor detection and segmentation. It is important to note that these results are based on the specific experimental setup and the particular dataset used. Different datasets or variations in preprocessing techniques and hyperparameters could potentially yield different performance outcomes. Therefore, further analysis, including evaluation on diverse datasets and consideration of additional performance metrics, would provide a more comprehensive understanding of the models' capabilities in brain tumor detection and segmentation.

### Postprocessing

After performing several experiments using, the UnetEfficientNet models with two heads for brain tumor classification and segmentation, we implemented post-processing techniques to further improve the outcomes. These techniques involved applying different thresholding methods to the images and refining the predicted values for both classification and segmentation tasks. In the case of classification, the post-processing involved thresholding the predicted values and analyzing the tumor size. Based on these criteria, the images were classified as either tumorous or non-tumorous, providing a more refined classification outcome. For the segmentation process, post-processing techniques included connected component labeling (CCL) along with thresholding and size analysis of the bounding boxes. By applying these techniques, the segmentation results were enhanced, resulting in improved accuracy and precision. Table 8 presents the results of the impact of post-processing on brain tumor segmentation in terms of threshold values and the size parameter. The models used for the experimentation include UnetEfficientnet-B0 to B5, and the tumor types analyzed are Meningioma, Glioma, and Pituitary. The table showcases the Dice scores obtained for each tumor type, along with the corresponding threshold values and sizes used. Additionally, the number of epochs and the global Dice score, representing the overall segmentation performance, are also included in the table.

**Table 8:** Impact of post-processing on brain tumor segmentation

Models	Tumor Types	DICE	Threshold	Size	Epoch	Global Dice
UnetEfficientnet-B0	Meningioma	0.96929	0.9	1200	30	91.35%
	Glioma	0.83345	0.65	1200		
	Pituitary	0.93788	0.65	100		
UnetEfficientnet-B1	Meningioma	0.97283	0.65	1200	30	93.21%
	Glioma	0.86621	0.9	1200		
	Pituitary	0.95737	0.9	100		
UnetEfficientnet-B2	Meningioma	0.88741	0.95	500	30	85.07%
	Glioma	0.77191	0.7	800		
	Pituitary	0.89278	0.85	1200		
UnetEfficientnet-B3	Meningioma	0.96412	0.8	1200	30	91.90%
	Glioma	0.84201	0.8	100		
	Pituitary	0.95101	0.95	100		
UnetEfficientnet-B4	Meningioma	0.98701	0.9	100	30	**94.03%**
	Glioma	0.87001	0.95	1200		
	Pituitary	0.96401	0.95	100		
UnetEfficientnet-B5	Meningioma	0.96977	0.6	1200	30	93.11%
	Glioma	0.85952	0.95	100		
	Pituitary	0.96411	0.9	100		

The influence of thresholding and size parameters in the post-processing technique for brain tumor segmentation contributes to enhancing the overall effectiveness of the model. The threshold parameter determines the pixel intensity value above which a pixel is considered part of the tumor region. By adjusting the threshold, we can control the sensitivity and specificity of the segmentation process. In the results, we can observe that different threshold values were chosen for each tumor type across the models. For example, lower thresholds of 0.6 and 0.65 were selected for Meningioma tumors in UnetEfficientnet-B5 and B1, respectively, while higher thresholds of 0.95 were used for Meningioma tumors in UnetEfficientnet-B2. The choice of threshold directly affects the segmentation accuracy. A higher threshold may be suitable for tumors with distinct and well-defined regions, while a lower threshold might be more appropriate for tumors with subtle or diffuse boundaries. The impact of thresholding can be observed in the Dice scores, where models with lower thresholds tend to achieve higher Dice scores, indicating better alignment between the predicted and ground truth tumor regions.

The size parameter controls the extent of the segmented tumor areas. It determines the minimum or maximum size of the connected components that are considered as tumor regions. In the results, we can see different size values selected for each tumor type in different models. For example, UnetEfficientnet-B0 utilized a size of 1200 for Meningioma and Glioma tumors, while UnetEfficientnet-B4 used a smaller size of 100 for Meningioma tumors. Choosing an appropriate size parameter helps filter out small noise or artifacts that might be erroneously detected as tumor regions, leading to more accurate segmentation results. A larger size threshold may be suitable for capturing larger tumor regions, while a smaller size threshold can help focus on more precise delineation of smaller tumor sub-regions. The impact of size can be observed in the Dice scores, where models with smaller size thresholds tend to achieve higher Dice scores, indicating better segmentation accuracy by filtering out small noise or artifacts. Overall, the choice of threshold and size parameters in the post-processing technique has a significant impact on the accuracy and reliability of brain tumor segmentation. By adjusting these parameters based on the characteristics of the tumor types and dataset, we can optimize the segmentation performance. A careful selection of thresholds and sizes ensures a balance between sensitivity and specificity, filtering out noise or artifacts while accurately delineating the tumor regions.

To evaluate the classification accuracy after post-processing, metrics such as precision, recall, and accuracy (Acc) were calculated. Precision measures the percentage of correctly classified tumorous cases among all predicted positive cases, recall measures the percentage of correctly identified tumorous cases among all actual positive cases, and accuracy represents the overall correctness of the classification. In terms of segmentation, the Dice score was used as a metric to assess the overlap between the predicted segmentation masks and the ground truth masks. The Dice score was calculated both before and after applying the post-processing techniques, allowing us to evaluate the improvement achieved through these techniques. Table 9 presents the results of the post-processing stage, including the Dice scores for segmentation and the precision, recall, and accuracy values for classification. These metrics provide insights into the effectiveness of the post-processing techniques in refining the classification and segmentation outcomes. Table 9 compares the performance of different post-processing techniques on brain tumor segmentation for various UnetEfficientnet models (B0 to B5). The results are presented in terms of Dice scores for the segmentation head, and precision, recall, and accuracy for the classification head.

**Table 9:** Performance comparison of post-processing technique in brain tumor segmentation

DL Models	Segmentation Head (DICE)		Classification Head		
	Without post processing	With post-processing	Precision	Recall	Accuracy
UNetEfficientnet-B0	89.47	91.35	96.67	97.00	94.80
UNetEfficientnet-B1	91.03	93.21	98.00	97.67	99.00
UNetEfficientnet-B2	82.73	85.07	80.33	81.67	93.00
UNetEfficientnet-B3	90.45	91.90	98.00	98.00	96.00
UNetEfficientnet-B4	**91.14**	**94.03**	**98.67**	**99.00**	**99.40**
UNetEfficientnet-B5	90.78	93.11	97.33	97.67	99.30

The analysis of the post-processing technique applied to the UnetEfficientNet models reveals its effectiveness in improving both the segmentation and classification outcomes of brain tumor analysis. For the segmentation task, the Dice scores were evaluated before and after the post-processing technique. Across all models, the post-processing technique resulted in higher Dice scores, indicating improved segmentation accuracy. For example, UnetEfficientNet-B0 achieved a Dice score of 89.47% without post-processing, which increased to 91.35% with post-processing. Similarly, UnetEfficientNet-B1 achieved a Dice score of 91.03% without post-processing, which improved to 93.21% with post-processing. This pattern was consistent across all models, indicating that the post-processing technique effectively refined the segmentation results. UnetEfficientNet-B4 demonstrates superior segmentation performance with a Dice score of 91.14% compared to UnetEfficientNet-B2, which achieved a lower Dice score of 82.73%. The higher Dice score of B4 indicates a higher degree of overlap between the predicted segmentation masks and the ground truth masks, signifying more accurate delineation of tumor regions. In contrast, B2 exhibits a lower level of segmentation accuracy.

In terms of classification, the precision, recall, and accuracy values were evaluated. The precision values represent the percentage of correctly classified tumorous cases among all predicted positive cases, while the recall values represent the percentage of correctly identified tumorous cases among all actual positive cases. The accuracy values represent the overall correctness of the classification. The post-processing technique improved the precision, recall, and accuracy values for most models, indicating better performance in classifying brain tumors. For example, UnetEfficientNet-B0 achieved a precision of 96.67%, recall of 97.00%, and accuracy of 94.8% without post-processing, which increased to 98.00%, 97.67%, and 99.0% respectively with post-processing. This improvement was consistent across most models, suggesting that the post-processing technique enhanced the classification accuracy. In terms of classification, UnetEfficientNet-B4 outperforms UnetEfficientNet-B2 with higher precision, recall, and accuracy values. B4 achieved precision, recall, and accuracy values of 98.67%, 99.00%, and 99.4%, respectively, while B2 had precision, recall, and accuracy values of 80.33%, 81.67%, and 93%, respectively. The higher classification metrics of B4 indicate a better ability to correctly classify tumorous cases, minimize false positives and negatives, and achieve overall higher accuracy in distinguishing between tumorous and non-tumorous brain regions.

The comparative analysis of the models reveals the varying degrees of improvement achieved through post-processing. UnetEfficientNet-B4 shows the highest improvement in both segmentation and classification, with the highest Dice score of 94.03% and precision, recall, and accuracy values of 98.67%, 99.00%, and 99.4%, respectively, after post-processing. UnetEfficientNet-B1 and B5 also demonstrate notable improvements in both segmentation and classification. On the other hand, UnetEfficientNet-B2 shows a lower improvement compared to other models, indicating the potential limitations of the post-processing technique in refining the results for certain architectural configurations. Overall, the UnetEfficientNet-B4 and UnetEfficientNet-B2 highlights the importance of architectural differences in model performance of best and worst performances among all. The superior performance of B4, compared to B2, in both segmentation and classification metrics underscores the significance of model architecture in achieving accurate and reliable results. The superior performance of UnetEfficientNet-B4 suggests that its architectural design, including the number of layers and parameters, is better suited for the task of brain tumor detection and segmentation.

By averaging the weight values of the best-performing models, we created an ensemble model to evaluate the segmentation outcome. This ensemble model, implemented through a weighted average method, yielded promising results in terms of the Dice score and Jaccard Index (IoU). The overall Dice score achieved by the ensemble model for brain tumor segmentation was 95.70%, which surpassed the individual performance of each utilized model.

**Fig. 22:**
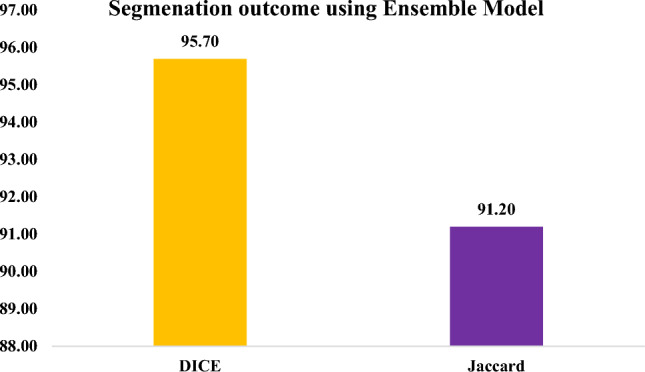
Brain tumor segmentation perfomance using Ensemble model

Additionally, the Jaccard Index (IoU) value obtained with the ensemble model was 91.20%. These results indicate that the ensemble model outperforms the individual models in terms of segmentation efficiency. It is important to note that applying the ensemble model requires conducting experiments with all the models to obtain their respective weight values. This process may be time-consuming and resource-intensive. However, the enhanced segmentation performance achieved by the ensemble model justifies the additional effort. Figure 22 presents the segmentation outcomes based on the Dice score and Jaccard Index using the ensemble model. These visualizations further illustrate the superiority of the ensemble model in terms of segmentation accuracy and efficiency.

**Fig. 23:**
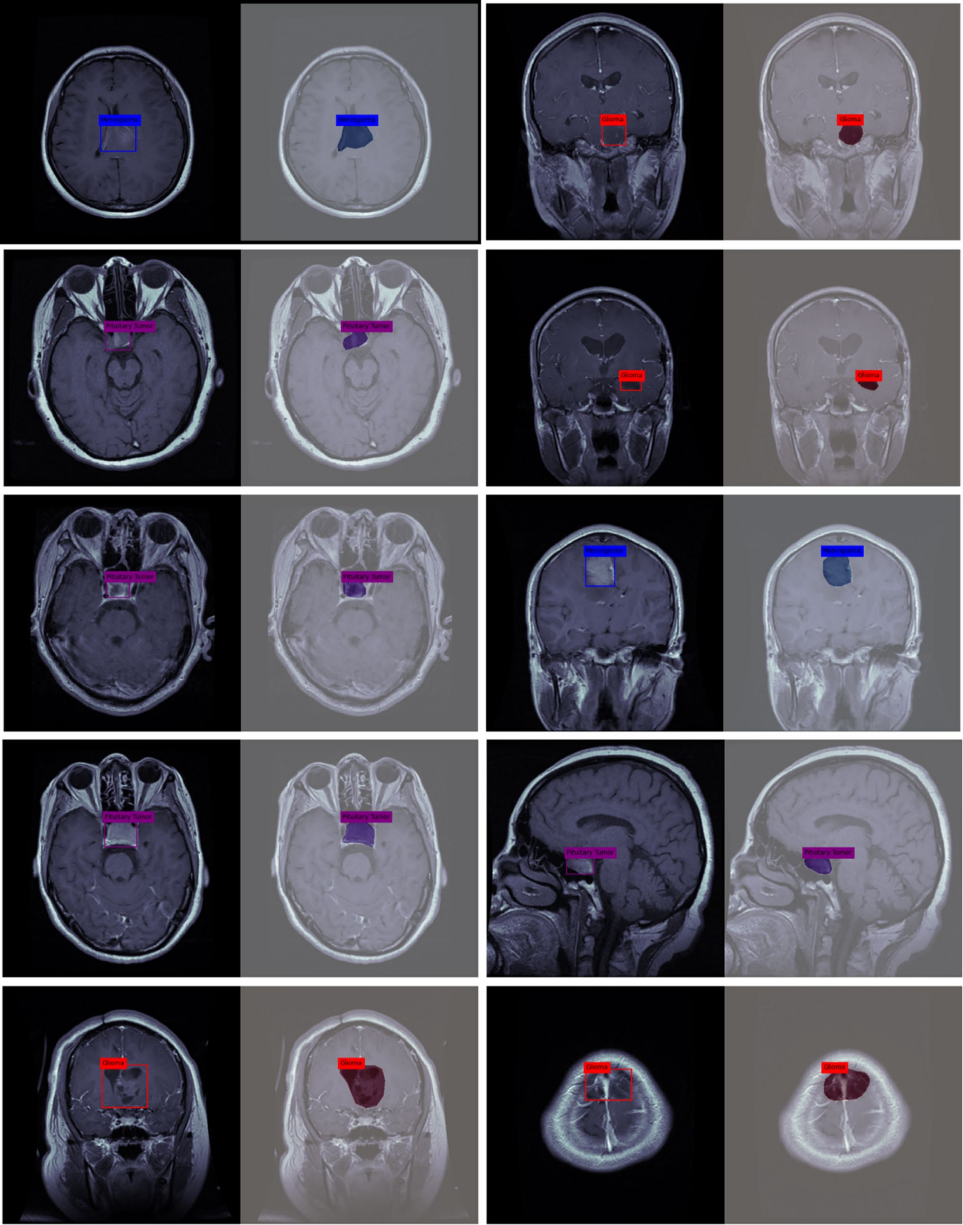
Classification and segmentation outcome for different types of brain tumors using UnetEfficientNet-B4 model (Left: Classification Head, Right: Segmentation Head)

The obtained sample outputs using the proposed model, with both the classification and segmentation heads, for all three types of brain tumors are presented in Fig. 23. The left side of the figure displays the classification outputs, showing the accurately classified tumors for each tumor type. On the right side, the segmented outputs are presented, highlighting the precise segmentation areas for each tumor type. The classification head of the model is responsible for accurately classifying the brain tumors into their respective types, namely Meningioma, Glioma, and Pituitary Tumor. The classification outputs on the left side of Fig. 23 demonstrate the model's ability to correctly identify and categorize the tumors. Meanwhile, the segmentation head of the model focuses on precisely delineating the tumor regions within the brain images. The segmented outputs on the right side of Fig. 23 showcase the model's effectiveness in accurately segmenting the tumors, providing valuable information about their spatial extent and location. To further visualize and validate the results, a video output has been prepared, providing a dynamic demonstration of the classification and segmentation outcomes. The link to the video can be accessed (https://youtu.be/kBXayUukUU8) for a comprehensive understanding and assessment of the model's performance in both classification and segmentation tasks.

## Discussion

In this study, we have presented the UnetEfficientNet-B4 model, which combines the Unet architecture with the EfficientNet models, for the detection and segmentation of brain tumors in MRI images. The model consists of two heads: a classification head for tumor detection and a segmentation head for precise delineation of tumor regions. By integrating both tasks into a single model, we aim to improve efficiency and accuracy in tumor analysis. Additionally, we have proposed a postprocessing technique using Connected Component Labeling (CCL) to further enhance the segmentation efficiency. To validate the performance of our model, we have employed ensemble techniques and compared the results with other state-of-the-art methods.

To validate the performance of our proposed model, we conducted experiments using the same dataset and compared the results with those obtained from five additional techniques (UnetEfficientNet-B0 to UnetEfficientNet-B5). This comparative analysis allowed us to assess the effectiveness and robustness of our model in relation to other state-of-the-art methods. By comparing our model's performance with the outcomes of recently published peer-reviewed articles, we were able to further validate the credibility and reliability of our study. This comparative analysis served as a benchmark for evaluating the effectiveness of our proposed model against established approaches in the field. This comprehensive validation, involving multiple techniques and comparison with published works, strengthens the reliability and significance of our proposed model. It provides further evidence of the model's capability to accurately detect and segment brain tumors, and it highlights its potential as a state-of-the-art solution in the field of medical image analysis.

In this work, we have presented the detailed comparative analysis of the proposed UnetEfficientNet-B4 model with various state-of-the-art methods for brain tumor detection and segmentation. The performance of each method is evaluated using metrics such as Dice score, Jaccard index, Precision, Recall, and Accuracy.

Upon analyzing (Table 10), we can observe that among the listed authors, only (Naser and Deen 2020), (Rai et al. 2021), (Sun and Wang 2021), (Rasool Reddy and Dhuli 2021), and (Sasank and Venkateswarlu 2022) have incorporated both detection and segmentation approaches in their studies. Notably, our previous work, as presented in (Rai et al. 2021), employed UnetResNext-50 for separate tumor segmentation and detection, achieving a remarkable Dice score of 95.73, which slightly outperforms our present work.

When considering all the utilized metrics including Dice score, Precision, Recall, and Accuracy, it becomes apparent that only our previous study (Rai et al. 2021) and (Rasool Reddy and Dhuli 2021) have employed all these metrics to validate the performance of their models. This comprehensive assessment allows for a more robust evaluation of the model's capabilities in terms of tumor segmentation and detection. By taking these factors into account, we can ascertain that our previous study, along with (Öksüz et al. 2021), has provided a more comprehensive evaluation by considering multiple metrics. This ensures a more thorough understanding of the model's performance in different aspects of tumor detection and segmentation. Furthermore, (Noreen et al. 2020) achieved an accuracy of 99.51% in classification by employing two models, Inception-v3 and DensNet201. It is important to note that they also performed only classification task on the same dataset as our approach, the figshare dataset, which comprised 3064 MRI images.

In contrast to the dataset utilization, (Huang et al. 2022) utilized the AFM-Net model and the BRATS-17 dataset, which comprised a substantial number of 10,517 images, the highest among all the studies mentioned. However, despite the large dataset size, they achieved an accuracy of only 92.10% in their classification task. This accuracy is significantly lower than the accuracy achieved in our present work. It indicates that the effectiveness of a model cannot solely rely on the dataset size, but also on the model architecture, optimization techniques, and other factors. Our proposed model, despite utilizing a smaller dataset, outperforms Huang et al. 2022) in terms of accuracy, demonstrating the efficacy of our approach in brain tumor classification.

After thorough validation of the proposed UnetEfficientNet-B4 model with state-of-the-art techniques listed in Table 10, we can conclude that our model achieves impressive results in terms of brain tumor segmentation and classification. The Dice score of 94.03% indicates a high degree of overlap between the predicted and ground truth segmentations, demonstrating the model's accuracy in delineating tumor regions. The Jaccard index of 98.67% further confirms the model's effectiveness in segmenting brain tumors, as it measures the similarity between the predicted and ground truth masks. In terms of classification performance, our proposed model achieves a Precision of 99.00%, indicating a low rate of false positives, and a Recall of 99.40%, indicating a low rate of false negatives. This showcases the model's capability to accurately classify tumor regions. Comparing our proposed model with other state-of-the-art methods, it outperforms several approaches in terms of segmentation performance, as evidenced by the high Dice score and Jaccard index. Furthermore, the ensemble segmentation approach, where multiple models are combined, achieves an overall Dice score of 95.70% and Jaccard index of 91.20%. This ensemble model demonstrates improved segmentation outcomes compared to individual models, highlighting the effectiveness of combining multiple models for better performance. Overall, the results obtained from our proposed UnetEfficientNet-B4 model, along with the ensemble segmentation approach, validate its superiority in terms of brain tumor segmentation and classification. The high accuracy, precision, and recall rates make it a promising solution for accurate and reliable brain tumor analysis.

**Table 10:** Performance comparison of the proposed model with state-of-art methods

Authors/Year	Methods	Dice (%)	Jaccard (%)	Pre (%)	Rec(%)	Acc (%)	MR Images	Database
Buda et al. (2019)	U-Net	82.00	–	–	–		3929	FLAIR MRI
Neelum Noreen et al. (2020)	Inception-v3	–	–	–	–	99.34	3064	Figshare
	DensNet201	–	–	–	–	99.51		
Siva Raja and rani (2020)	DAE + JOA + SoftMax regression	–	–	96.00	–-	98.5	274	BRATS-15
Çinar and Yildirim (2020)	Hybrid CNN Architecture	–	–	–	94.70	97.01	253	Kaggle
Naser and Deen (2020)	DL-TL	84.00	–	–	92.00	92.00	3929	FLAIR MRI
Rai et al. (2021)	UnetResNext-50	95.73	86.00	90.12	89.70	99.7	3929	FLAIR MRI
Sun and Wang (2021)	ASCNN	79.73	–	–	80.45		285	BRATS-18
Öksüz et al. (2021)	ResNet18 + ShallowNet + SVM	–	–	–	95.27	97.25	3064	Figshare
Ahmad and Choudhury (2022)	VGG19 + SVM	–	98.63	99.51	99.27	99.39	250	Kaggle
Rasool Reddy and Dhuli (2021)	ELDP + SVM	83.14	–-	95.53	89.17	94.44	274	BRATS-15
Huang et al. (2022)	AFM-Net	–	–	–	98.30	98.10	2686	SHCMU
		–	–	–	91.70	92.10	10,517	BRATS-17
Sasank and Venkateswarlu (2022)	FrCN + MSFO	95.24	90.90	–	95.23	97.01	494	BRATS-20
		92.30	85.71	–	92.31	95.56	335	BRATS-19
		93.33	87.50	–	87.50	95.23	266	BRATS-18
Ramtekkar et al. (2023)	Whale + CNN	–	–	98.00	100.0	98.90	253	Kaggle
Saeedi et al. (2023)	2D CNN	–	–	94.75	95.75	96.47	3264	Kaggle
Mahmud et al. (2023)	CNN	–	–	91.19	–-	93.30	3264	Kaggle
Patil and Kirange ( 2023)	Ensemble Deep CNN (EDCNN)	–	–	96.66	98.30	97.77	3064	Figshare
Krishnapriya and Karuna (2023)	VGG-19	–	–	–	–	99.48	253	Kaggle
Proposed	**Two-Headed UnetEfficientNetB4 + Post Processing**	94.03	–	98.67	99.00	99.40	3064	Figshare
	**Ensemble (Segmentation)**	**95.70**	91.20	–	–	–		

*FrCN* Fully resolution convolutional network, *MSFO* Modified Sunflower Optimization, *ASCNN* Application-Specific CNN, *ELDP* Entropy-based local directional pattern, *AFM-Net* Adaptive multisequence fusing neural network, *SHCMU* Shengjing Hospital of China Medical University, *DL-TL* Deep Learning and Transfer Learning

## Conclusion

This paper presented the automatic segmentation and detection of brain tumors from MRI images using a two-headed UnetEfficientNet-B4 model. The study utilized the Figshare dataset, consisting of 3064 images categorized into three tumor classes: Meningioma, Glioma, and Pituitary for experimentation with the proposed model. The proposed model combined EfficientNet models with a modified two-headed Unet architecture, enabling parallel detection and segmentation tasks. For validation of the proposed model, experiments were performed with six models, UnetEfficientNet-B0 to UnetEfficientNet-B5, including the proposed model. A unique approach was applied to obtain the outcome in two heads, the segmentation and classification heads. The post-processing technique using CCL was also implemented to further improve the segmentation outcome. Additionally, the ensemble technique was tested to validate the overall segmentation model performance. The performance evaluation of all the models was done using five parametric evaluation metrics: Jaccard Index, DICE score, accuracy, precision, and recall. The proposed UnetEfficientNet-B4 model was also compared with state-of-the-art methods and achieved an excellent accuracy of 99.4% after post-processing. Furthermore, it produced a high Dice score, precision, and recall of 94.03%, 98.67%, and 99.00%, respectively, after post-processing. The global Dice and Jaccard index values of 95.70% and 91.20% were achieved using the ensemble technique. The proposed UnetEfficientNet-B4 model proved to be very efficient and accurate for the automatic and parallel detection and segmentation of brain tumors from MRI images.

Although the suggested model performed well with high accuracy, there is still room for improvement. In future work, the use of GANs (generative adversarial networks) will be explored to generate synthetic data samples of minority classes to enhance the total number of data samples. Additionally, the inclusion of 3D models and datasets will be considered for better visualization and further scalability of the present study.

### Supplementary Information

Below is the link to the electronic supplementary material.

Supplementary file1 (MP4 11332 KB)

## Data Availability

The dataset used for experimentation in this study can be accessed publicly at the following link: https://www.kaggle.com/datasets/ashkhagan/figshare-brain-tumor-dataset.
